# West Nile virus spread in Europe: Phylogeographic pattern analysis and key drivers

**DOI:** 10.1371/journal.ppat.1011880

**Published:** 2024-01-25

**Authors:** Lu Lu, Feifei Zhang, Bas B. Oude Munnink, Emmanuelle Munger, Reina S. Sikkema, Styliani Pappa, Katerina Tsioka, Alessandro Sinigaglia, Emanuela Dal Molin, Barbara B. Shih, Anne Günther, Anne Pohlmann, Ute Ziegler, Martin Beer, Rachel A. Taylor, Frederic Bartumeus, Mark Woolhouse, Frank M. Aarestrup, Luisa Barzon, Anna Papa, Samantha Lycett, Marion P. G. Koopmans

**Affiliations:** 1 Roslin Institute, University of Edinburgh, Edinburgh, United Kingdom; 2 Usher Institute, University of Edinburgh, Edinburgh, United Kingdom; 3 Erasmus MC, Viroscience and Pandemic and Disaster Preparedness Centre, Rotterdam, the Netherlands; 4 Department of Microbiology, Medical School, Aristotle University of Thessaloniki, Thessaloniki, Greece; 5 Department of Molecular Medicine, University of Padova, Padua, Italy; 6 Institute of Diagnostic Virology, Friedrich-Loeffler-Institut, Greifswald-Riems, Germany; 7 Institute of Novel and Emerging Infectious Diseases, Friedrich-Loeffler-Institut, Greifswald-Riems, Germany; 8 Department of Epidemiological Sciences, Animal and Plant Health Agency, United Kingdom; 9 Centre for Advanced Studies of Blanes (CEAB-CSIC), Girona, Spain; 10 Centre for Research on Ecology and Forestry Applications (CREAF), Barcelona, Spain; 11 Catalan Institution for Research and Advanced Studies (ICREA), Barcelona, Spain; 12 Research Group for Genomic Epidemiology, Technical University of Denmark, Kongens Lyngby, Denmark; New York Medical College, UNITED STATES

## Abstract

**Background:**

West Nile virus (WNV) outbreaks in birds, humans, and livestock have occurred in multiple areas in Europe and have had a significant impact on animal and human health. The patterns of emergence and spread of WNV in Europe are very different from those in the US and understanding these are important for guiding preparedness activities.

**Methods:**

We mapped the evolution and spread history of WNV in Europe by incorporating viral genome sequences and epidemiological data into phylodynamic models. Spatially explicit phylogeographic models were developed to explore the possible contribution of different drivers to viral dispersal direction and velocity. A “skygrid-GLM” approach was used to identify how changes in environments would predict viral genetic diversity variations over time.

**Findings:**

Among the six lineages found in Europe, WNV-2a (a sub-lineage of WNV-2) has been predominant (accounting for 73% of all sequences obtained in Europe that have been shared in the public domain) and has spread to at least 14 countries. In the past two decades, WNV-2a has evolved into two major co-circulating clusters, both originating from Central Europe, but with distinct dynamic history and transmission patterns. WNV-2a spreads at a high dispersal velocity (88km/yr–215 km/yr) which is correlated to bird movements. Notably, amongst multiple drivers that could affect the spread of WNV, factors related to land use were found to strongly influence the spread of WNV. Specifically, the intensity of agricultural activities (defined by factors related to crops and livestock production, such as coverage of cropland, pasture, cultivated and managed vegetation, livestock density) were positively associated with both spread direction and velocity. In addition, WNV spread direction was associated with high coverage of wetlands and migratory bird flyways.

**Conclusion:**

Our results suggest that—in addition to ecological conditions favouring bird- and mosquito- presence—agricultural land use may be a significant driver of WNV emergence and spread. Our study also identified significant gaps in data and the need to strengthen virological surveillance in countries of Central Europe from where WNV outbreaks are likely seeded. Enhanced monitoring for early detection of further dispersal could be targeted to areas with high agricultural activities and habitats of migratory birds.

## Author summary

Evidence for the drivers for West Nile virus (WNV) dispersal has been not clear in Europe. Here, we have comprehensively described the dispersal history of the currently predominant WNV lineage in Europe and estimated the contribution of key drivers of spread between and within countries. By fitting several phylodynamic and phylogeographic models, we found that 1) WNV in Europe has a greater lineage diversity than in other regions of the world. 2) Agricultural intensity had the greatest impact on both WNV spread direction and velocity. 3) WNV spread direction was specifically associated with urbanization and bird habitats. 4) Climate change and bio-diversity changes predicted viral genetic diversity over time. Our study revealed that to enhance preparedness for potential outbreaks, it is important to further define these drivers, and increase sampling for WNV in areas that appear to be a source of WNV in other countries in Europe, as well as in regions with known risk factors but where WNV has not yet been detected.

## Introduction

Mosquito-borne viruses are a considerable public health problem worldwide, causing infections in both humans and animals [1]. For the European region, West Nile virus (WNV) is one of the mosquito-borne viruses which can cause severe disease in humans and has been increasing in prevalence and geographic range over the past decade [2]. WNV belongs to the family *Flaviviridae* (genus *Flavivirus*) with an enveloped, single-stranded RNA genome [3]. The transmission cycle of WNV involves mosquitoes (mainly of the *Culex* species) as vectors and birds as amplifying reservoir hosts [4], while humans and other mammals are considered dead-end hosts [1]. Dead-end hosts are not thought to contribute significantly to transmission in the natural life cycle of the virus. However, for humans, the potential for virus transmission through blood transfusion and organ transplantation has impacted blood and transplantation donor programs, with mandatory screening introduced in regions where exposure to WNV is possible.

Currently, nine distinct lineages (WNV-1 to WNV-9) of WNV have been identified globally, yet little is known about their phenotypic properties [5,6]. WNV-1 and WNV-2 strains have been identified most often in human and animal cases on multiple continents, while strains within WNV-3 to WNV-9 have been detected from mosquitoes, birds, equines, and amphibians [5,7]. The lineage responsible for the majority of WNV outbreaks in Europe in recent years is WNV-2, although cases of WNV-1 have also been recently identified [8,9]. In contrast, only WNV-1 is circulating in North America, resulting from a single introduction of the virus into the continent [10].

WNV circulation in Europe was first reported in the 1960s [11]. Since 1996, an increasing number of WNV outbreaks in humans and equines have been detected in Southeast and Central Europe [12], progressively expanding to previously non-endemic areas with a notable northward trend [13]. In addition, available data highlight considerable differences between successive years, with, for instance, particularly severe regional WNV outbreaks involving both humans and equids in Italy, Serbia, and Greece [8,14,15], indicating multiple complex factors may have impact on the virus’s spread and emergence. Understanding which factors driving the genetic diversity and spread of WNV in Europe is crucial for effective surveillance and control strategies.

Previous phylogeographic studies on the prevalence and spread of WNV in European countries [16–19] used data up to 2019. These studies focused specifically on Germany [16], Italy [18] or Greece [17], with limited Europe-wide geographical coverage or a lack of precise sampling location data. Also, the restriction to the user of whole genomes only reduced the size of the dataset considerably, as success of WGS with surveillance samples is variable. These studies performed between country/region phylogeographic analyses, and we extend this by using integrated advanced phylodynamic and statistical models, risk factor analysis, and coordinated datasets encompassing virus genomes (whole genomes and partial genome sequences) and metadata from sampling humans, (wild) animals, and vectors from 2005–2021. Moreover, none of these studies have delved into the significant shifts in the epidemiology and ecology of WNV resulting from its northward migration, which raises novel questions regarding this expansion, for instance, the further genetic changes of the viruses and factors influencing the direction of viral spread and whether the virus might extend into previously uninfected regions in the north, such as the United Kingdom.

Our research provides a comprehensive and robust analysis of WNV dynamics in Europe. We describe the evolution and genetic diversity of WNV in Europe by conducting a collaborative effort, supported by the VEO consortium (https://www.veo-europe.eu/). The consortium aims to explore potential applications of complex data mining approaches using combined datasets from a multitude of (open) sources. Focusing on a predominant lineage that has spread widely in Europe in recent years, we further explored the dispersal history in regions with yearly outbreaks and in regions with sporadic outbreaks and assessed the possible role of different environmental drivers in the spread of the virus. A potential risk factor dataset was compiled from experts in the ecology of the hosts and pathogens. The phylodynamic models traced the spatio-temporal spread of different WNV lineages, and we identified key geospatial drivers from the potential risk factor data set, as well as locating areas requiring targeted surveillance. Our results suggest that—in addition to ecological conditions favouring bird- and mosquito- presence—agricultural land use may be a significant driver of WNV emergence and spread. Our study also identified significant gaps in data and the need to strengthen virological surveillance in specific countries and regions.

## Materials and methods

### Methods overview

In this study, we generate a unique dataset of historical and new virus genome sequences, including the two sub-lineages of WNV-2 (WNV-2a and WNV-2b) and several other strains. The complete evolution, transmission and spread history of the recent predominant WNV-2a in Europe was studied using comprehensive phylodynamic analysis based on viral genome sequence data and detailed epidemiology data. Spatially explicit phylogeographic models were then fitted to the WNV sequences with a collection of well-assessed environmental and ecological data, to uncover important drivers of viral dispersal direction and dispersal velocity. Furthermore, a skygrid-generalised linear model (GLM) was employed to evaluate the relationship between changes in temperature and biodiversity and variations in viral genetic diversity during the last two decades.

### Genome sequence data collection

We generated new previously unpublished WNV-2 genomes from samples collected from Italy (n = 8) and the Netherlands (n = 6) between 2019 and 2020. Virus isolation, identification and sequencing are as previously described [8,20,21]. Sequences have been submitted to GenBank with accession IDs OP561452-OP561459, OP762592-OP762597 (S1 Table).

Apart from the new WNV genomes obtained in this study, we downloaded all available nucleotide sequences of WNV isolated from Europe as of 02 June 2022 from NCBI (www.ncbi.nlm.nih.gov). To identify possible cross-continent transmission, we BLAST our European WNV dataset against sequences database at NCBI using Geneious Prime 2021.1.1 to find closely matching genomes in other continents (https://www.geneious.com). We counted in total 485 WNV sequences (from unique samples), including 226 full genome sequences over a 50-year span from 1971 to 2021. Metadata of WNV sequences was updated by the aforementioned European collaborative consortium. For example, we have adjusted the travel history of human cases (if known) to locate the original source of infection (S1 Text: Genome Sequence data collection).

### Phylodynamic reconstructions

We applied multiple phylogenetic and phylodynamic models to map the evolution and spread history of WNV in Europe. We generated preliminary phylogenetic trees using a maximum likelihood model using IQTree [22] and assessed the temporal signal of the sequence data using TempEst v1.5 [23]. We reconstruct time-scaled phylogenies of WNV-2a, the main sub-lineage identified in Europe, using Bayesian phylogenetic methods in BEAST version 1.10.4 [24]. We generated separate original time-scaled trees (data of whole genome sequence, NS5 gene and NS3 gene) first and conducted phylogeographic analysis using empirical trees. We quantified the between-country and within-country transmissions by combining the phylogenies of two distinct genes [24]. We used an asymmetric model and incorporated the Bayesian Stochastic Search Variable Selection procedure (BSSVS) to identify a sparse set of transmission rates which reflect the statistically supported connectivity [25]. We also estimated the expected number of transmissions (jumps) between countries and within countries using Markov rewards [26]. The discrete trait analysis serves our study with multiple key objectives. It quantifies between-country transmissions, identifying primary donor countries from where WNV strains are seeded within Europe, and providing insights into potential routes of introduction and spread. Mapping the transmission network geographically allows us to compare it with country-specific management and surveillance efforts, assessing the effectiveness of existing strategies in controlling WNV spread. Additionally, the joint analysis of NS3 and NS5 sequence datasets ensures better geographical coverage and a more comprehensive transmission history. In addition, a continuous phylogenetic diffusion model with a Relaxed Random Walk extension [27] was further applied to explore the geographical spread of WNV. The results presented in the main text primarily utilize NS3 data, which has broader geographic coverage and a larger sample size. The continuous model results serve as validation for the findings obtained using NS3 data. More detailed methodologies were described in the S1 Text (see section Phylodynamic reconstructions).

### Spatial and temporal predictors of WNV dispersal

#### Collection of predictor data indicating drivers of disease emergence and/or spread

We collected a list of potential predictors that are thought to be associated with viral dispersal (n = 37) as well as time-varied predictors that may correlate with viral genetic diversity variations over time (n = 22). Data sources, original resolutions, along with definitions are provided in the S2 and S3 Tables. Predictors were mainly assigned into five categories, including 1) climate and weather, 2) land use and cover, 3) topography, 4) socio-economic factors and 5) biodiversity (S2 Table). More details are provided in the (see section Collection of predictor data indicating drivers of disease emergence and/or spread). We evaluated the data quality of the predictor datasets against six characteristics (Accuracy & precision, Reliability & consistency, Timeliness, Completeness, and Availability & accessibility, Granularity) using a newly developed protocol [28], which revealed that the data sources were of very good quality, scoring highly for accuracy, reliability, and availability (S2 Table).

#### Identification of drivers on viral presence and dispersal

We tested the associations between the above set of potential predictors of presence (S1 Fig) and dispersal of WNV using R package “seraphim” [29], which has been developed to study the time-scaled phylogeography in an environmental context; it extracts the spatio-temporal information from phylogenetic trees and uses this information to calculate and plot dispersion statistics.

We first tested the association of the dispersal directions for each branch of WNV in the phylogenetic tree (from an ancestral node to its descendants), with the paths that correspond to the environmental value changes through the predictor maps. We tested if the virus tended to remain in areas with lower/higher environmental values (local establishment), and/or the tendency of the lineages to disperse towards lower/higher values of the predictive factors (spread), by estimating the Bayes factor (BF) comparing values explored under the inferred model with the null dispersal model simulated along the tree [29]. It should be noted that the impact on dispersal direction should be evaluated with caution because it is significantly impacted by sampling and should be viewed as a more descriptive exploration than a definite statistical test [30].

We also tested the effect of the same set of predictors on viral dispersal velocity (i.e., how fast the virus spread and how long it took to spread), by examining the relationship between environmental distances and lineage dispersal durations, calculated from predictor rasters, using the "least-cost" path model [31] and the "CIRCUITSCAPE" model [32] (please see S1 Text: Prediction of drivers on viral dispersal velocity).

#### Identification of drivers on changes of viral population diversity over time

We applied the skygrid-GLM model [33] (an extension of the skygrid coalescent model that integrates external covariates in a generalized linear model framework) to jointly infer the WNV effective population size along with the coefficient that relates it to the time-varied predictors (n = 22) (S3 Table and S2 Fig). Here we examine the temporal relationship between the effective population size of WNV and the data describing climate-, land-use- and bio-diversity changes in Europe in 2004–2021 (defined by analysing the datasets described in the S3 Table and S2 Fig).

## Results

### Diverged WNV lineages found in Europe

We found a positive correlation between cumulative human cases in the European Union (EU)/ European Economic Area (EEA) reported by the European Centre for Disease Prevention and Control (ECDC, between 2008–2021, total n = 4188) and the number of WNV-2 sequences (between 2004–2021, total n = 485) provided from different countries (correlation coefficient = 0.75, p<0.005) (S3A Fig). Among the 22 European countries that have human cases reported, 15 have sequences available, including Greece, Italy, Romania, Serbia, Hungary, Spain, Austria, France, Bulgaria, Germany, Netherlands, Slovenia, Portugal, Slovakia and Czech Republic (Czechia); sequences are missing from seven countries (Croatia, Kosovo, Albania, Bosnia and Herzegovina, North Macedonia, Montenegro, and Cyprus) in the east region (S3B Fig).

**Fig 1:**
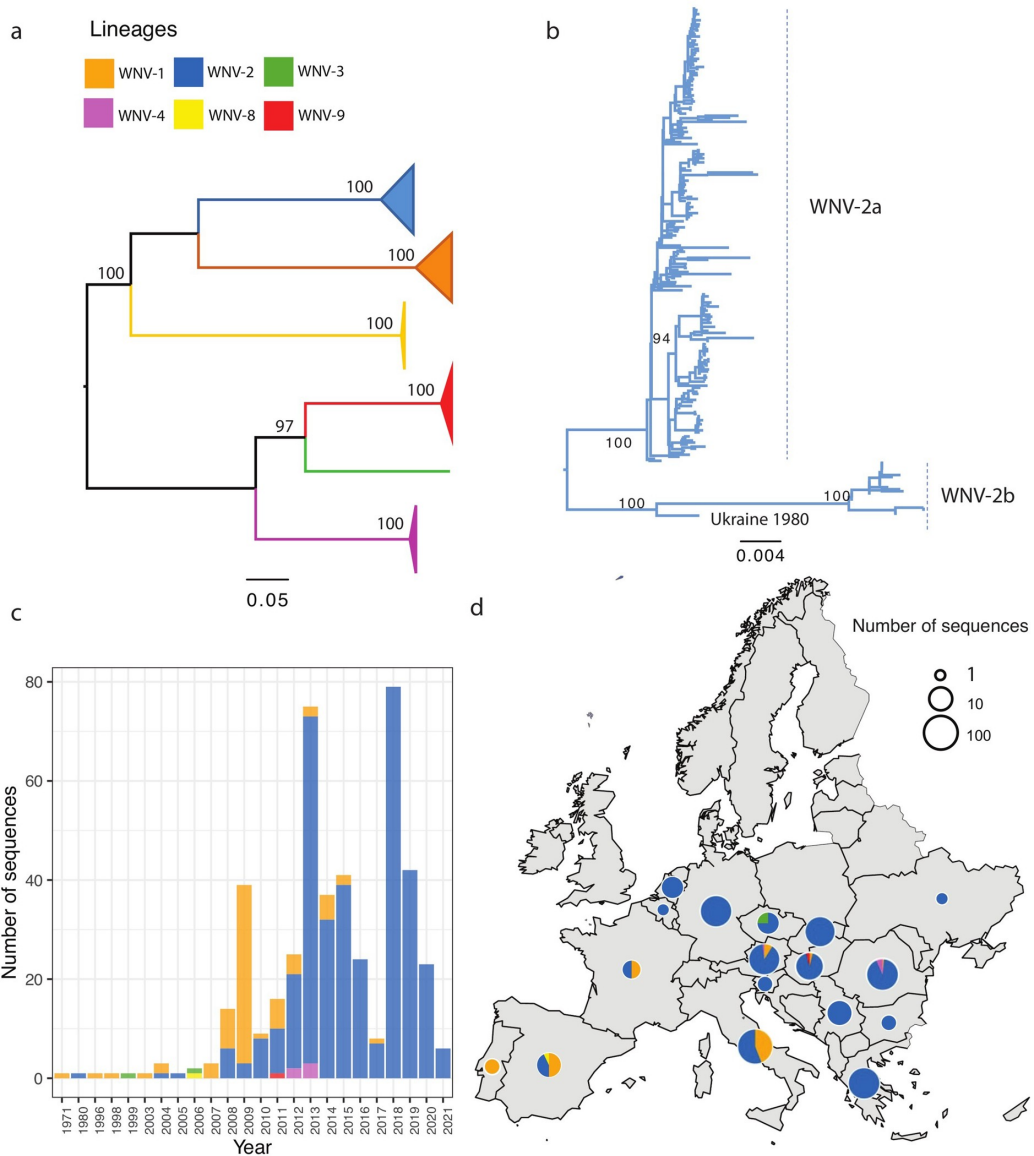
Phylogenetic analysis of WNV full and partial nucleotide sequences detected from Europe. The evolutionary distances were computed using the optimal GTR+I model, the phylogenetic tree was constructed with the Maximum likelihood (ML) method. Bootstrap values are given for 1000 replicates. (a) ML tree of all lineages found in Europe. The branches of lineages are all collapsed and shown as rectangles; (b) The subtree of WNV-2 sequences; (c) The WNV lineages distribution over time using the same color showing on the tree; (d) The geographical distribution of WNV lineages. Map with a small pie chart showing the total number of sequences detected (on a logarithmic scale) per country, with each slice proportional to the number of distinct WNV lineages within that country. The European shapefile used in the study was obtained from Data and Maps for ArcGIS (formerly Esri Data & Maps, https://www.arcgis.com/home/group.html?id=24838c2d95e14dd18c25e9bad55a7f82#overview) under a CC-BY 4.0 license.

The maximum likelihood tree of all available WNV genome sequences showed the phylogenetic relationships among the six lineages (out of the total nine lineages globally [6]) which have been detected in Europe [7] (Fig 1). Among the six lineages, WNV-2 had the largest number of sequences available, accounting for 82% of all publicly shared WNV sequences in Europe so far. The earliest WNV-2 genome (JX041631.1) was sampled from birds in Eastern Europe (Ukraine) in 1980 (Fig 1B and 1C). The dominant sub-lineage WNV-2a emerged in 2004 and became the dominant lineage in the past ten years, while the other lineages were rarely seen in the same time period. In addition, there was a separate small sub-lineage WNV-2b composed of sequences mainly from Romania, Italy, and Russia in 2011–2015, and one detection in Greece in 2018 [34] (Figs 1B and S4).

In comparison, sequences of the second-largest lineage (WNV-1) have been found in seven European countries (Austria, Italy, Spain, France, Hungary, Romania, and Portugal) since 1971. Most WNV-1 sequences were reported in Italy (72% of total WNV-1 sequences). A small number of WNV-1 genomes have been collected from human samples, including nine from Italy between 2009 and 2013 and four from Spain in 2020. WNV sequences belonging to lineages 3, 4, 8, and 9 were only sporadically reported: WNV-3 strains were only found in Czech Republic in 1997 and 2006; WNV-4 in Romania in 2012–13; WNV-8 was only found in Spain in 2006, while WNV-9 genomes were obtained from Austria in 2013 and Hungary in 2011 (Figs 1C and 1D and S4). In addition, up to 2021, these lineages (WNV 3, 4, 8 and 9) were only collected from non-human hosts (mainly birds or mosquitoes including amphibian feeding species of mosquitoes, and very few equines) [35].

### Phylodynamics of predominant sub-lineage WNV-2a

Given the potential risk of human infection, it is important to monitor and study all lineages of WNV circulating in mosquitoes. Considering the distinct genetic diversity, the number of sequences available, and the dominance of WNV-2 as a cause of human disease, the detailed phylodynamic analyses were focused on WNV-2a. These sequences were collected from 14 countries between 2004 and 2021, with most sequences from Greece (n = 64), Italy (n = 50), and Germany (n = 46) (S1 Table). The sequences were collected from six host types, with 30% from birds [including predatory birds (35%), songbirds (20%), captive birds (20%) and others], 30% from mosquitoes, and 40% from humans and other mammals (S5 Fig).

**Fig 2:**
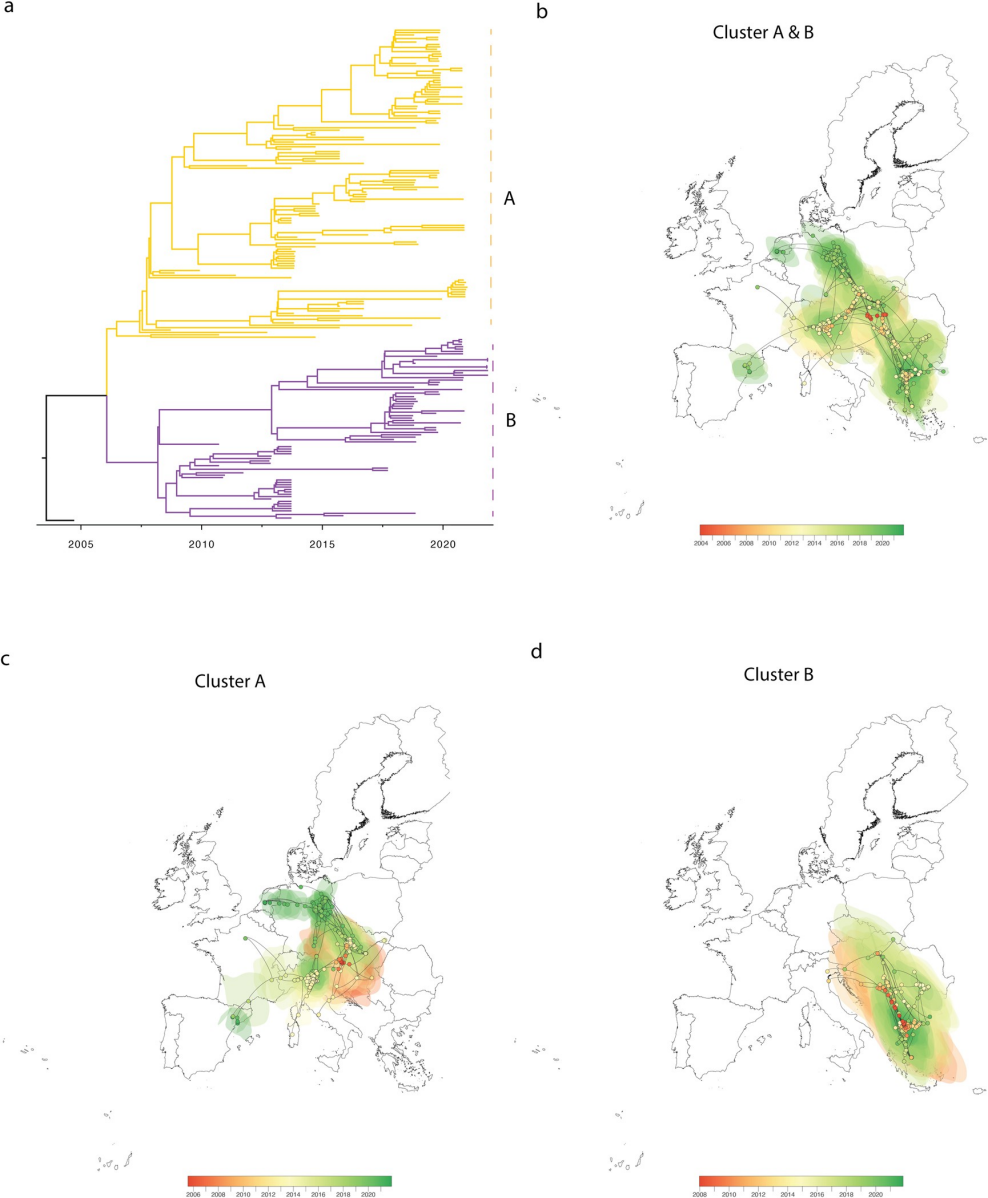
Time-scaled phylogeny of WNV-2a genomes in Europe. (a) Time-scaled MCC (maximum clade credibility) tree of WNV full genome sequences isolated in Europe (n = 192), the two clusters A and B are labelled on the right. A distinct phylogeographic analysis has been based on the NS3 gene by continuous phylogeographic inference based on 1,000 posterior trees. Spatiotemporal diffusion of all WNV- 2a in Europe (b), of Cluster A (c) and Cluster B (d). These MCC trees are superimposed on 80% of the highest posterior density (HPD) interval reflecting phylogeographic uncertainty. Nodes of the trees, as well as HPD regions, are colored by timescale from red (the time to the most recent common ancestor, TMRCA) to green (most recent sampling time), and the oldest nodes (and corresponding HPD regions) are here plotted on top of youngest nodes. The European shapefile was created using the R package “raster” (https://cran.r-project.org/web/packages/raster/).

The sequences of WNV-2a found in Europe exhibited considerable genetic divergence from those found in other continents, e.g., with a time to the Most Recent Common Ancestor (TMRCA) exceeding 40 years compared to sequences from Africa. As a result, in our subsequent continental-scale phylogeographic analysis, we treated the WNV-2a clade as a unique introduction into Europe. The time-scaled phylogeny of full genome sequences of WNV-2a is rooted with the first WNV-2a identified in Europe (in Hungary in 2005), with the estimated TMRCA dated back to July 2003 (95% highest posterior density (HPD) interval: November 2002 to July 2004, Fig 2A). The WNV-2a sequences in Europe were split into two distinct sub-clusters (A and B) on the tree composed of full genome sequences, which were previously named the central-eastern clade and southeastern clade, respectively [16]. Further, the phylogeographic analysis reconstructed the spread history of the WNV-2a sub-lineage over the past 20 years from the time-scaled phylogenies, which showed the different trajectories of Cluster A and B: Both Cluster A and Cluster B are present in Serbia, Slovakia, Hungary, Slovenia, and Italy. Cluster A has also been transmitted to Austria, the Czech Republic, Germany, the Netherlands, France, and Spain, while Cluster B has also been found in Greece, Romania, and Bulgaria (Fig 2B–2D).

**Fig 3:**
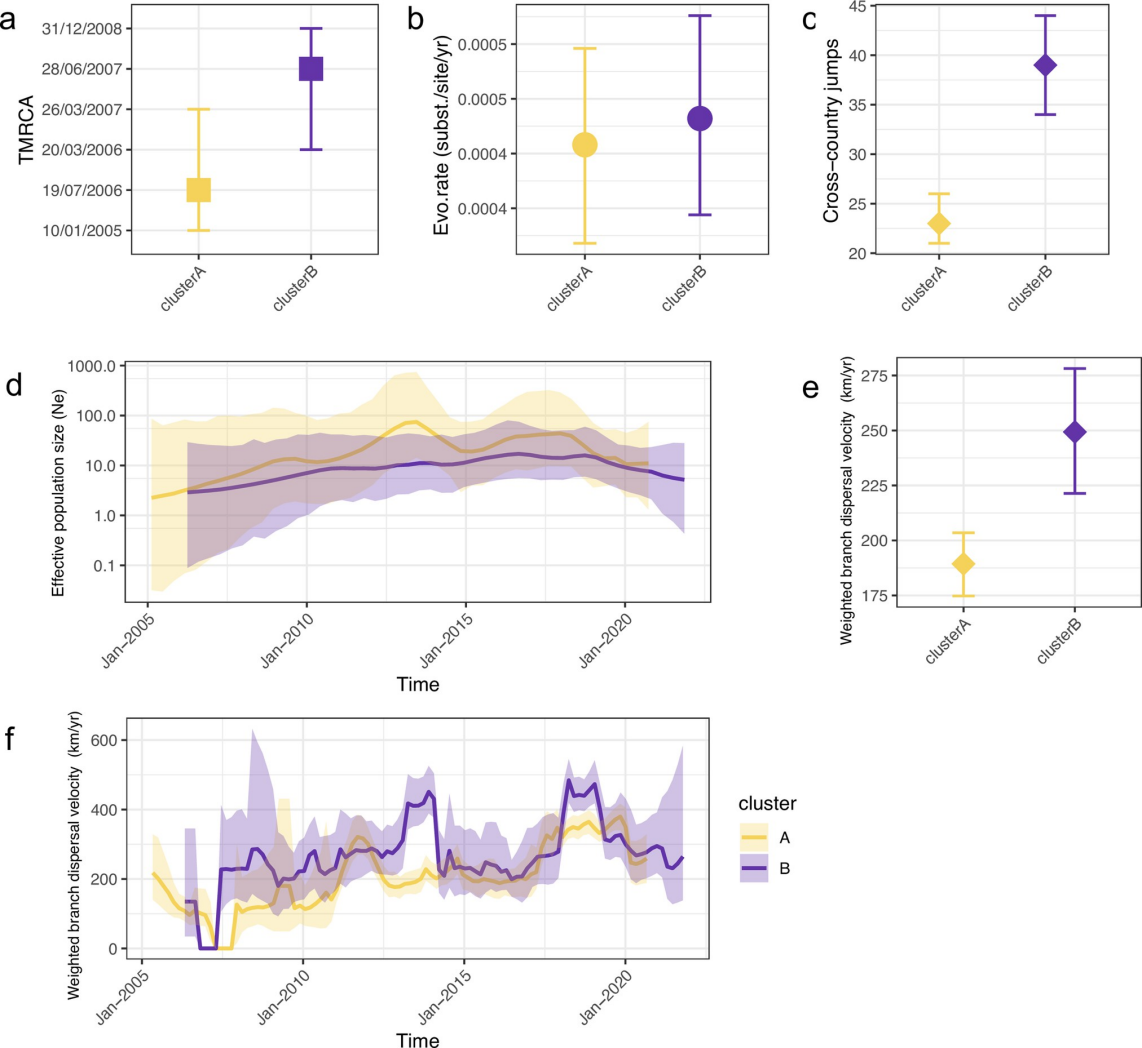
Comparisons of two co-circulating clusters of WNV-2a in Europe. (a) The mean time of the most recent ancestor (TMRCA) and 95%HPD interval for each cluster. (b) The mean clock rate (substitutions per site per year, subst./site/yr) and 95% HPD interval for each cluster was estimated using an uncorrelated relaxed molecular clock model. (c) The Mean number of Markov jump between countries and 95%HPD interval for each cluster were estimated using a continuous-time Markov chain (CTMC) model. (d) Estimation of effective population size via time and a 95% HPD interval using the Skygrid coalescent model. The logarithmic effective number of infections (Ne) vs. viral generation time (t), representing effective transmissions is plotted over time. (e) The mean of weighted dispersal velocity averaged over the branches of the entire tree (km/yr) and (f) The weighted dispersal velocity over time (km/yr) with a 95% HPD interval estimated using the continuous phylogenetic diffusion model.

We estimated the time of emergence and rate of evolution, as well as the population dynamics and speed of spread for the two WNV-2a clusters A and B. WNV of Cluster A emerged in approximately July 2006 (with 95% HPD between January 2005 and March 2007; Fig 3A). The estimated ancestor country of Cluster A was Austria, with subsequent sequences identified mainly in Germany and Italy (Fig 2). With a similar evolution rate, Cluster B emerged later in June 2007 (with 95% HPD between March 2006 and December 2008) from Hungary, and most subsequent sequences were collected from Greece (Figs 2 and 3A and 3B).

We also compared the changes of effective population size (Ne) of the two clusters. The effective population size of cluster A peaked around 2014, which corresponded to the expansion phase of the epidemic, and then decreased slightly till the second peak at around 2018, which was consistent with a high WNV activity in multiple regions. In comparison, there were fewer substantial changes in Ne of Cluster B (Fig 3D).

The estimations of the weighted dispersal velocity of WNV-2a in Europe vary between 88 km/yr (full genome), 215 km/yr (NS3 gene), and 180 km/yr (NS5 gene), respectively. The dispersal velocity of Cluster B sequences was higher (mean of 249 km/yr with 95% HPD between 221 and 278 km/yr using NS3 data) than those of Cluster A (mean of 189 km/yr with 95% HPD between 175 and 203km/yr using NS3 data) (Fig 3E and 3F). This finding is consistent with the estimation of frequencies of between-countries transmissions: WNV of Cluster B tended to jump more frequently among countries, with an overall number of 39 jumps (95% HPD between 34 and 44)—almost twice more often than the number of jumps observed for Cluster A (Fig 3C).

### Quantified transmission frequencies between European countries and within Greece

**Fig 4:**
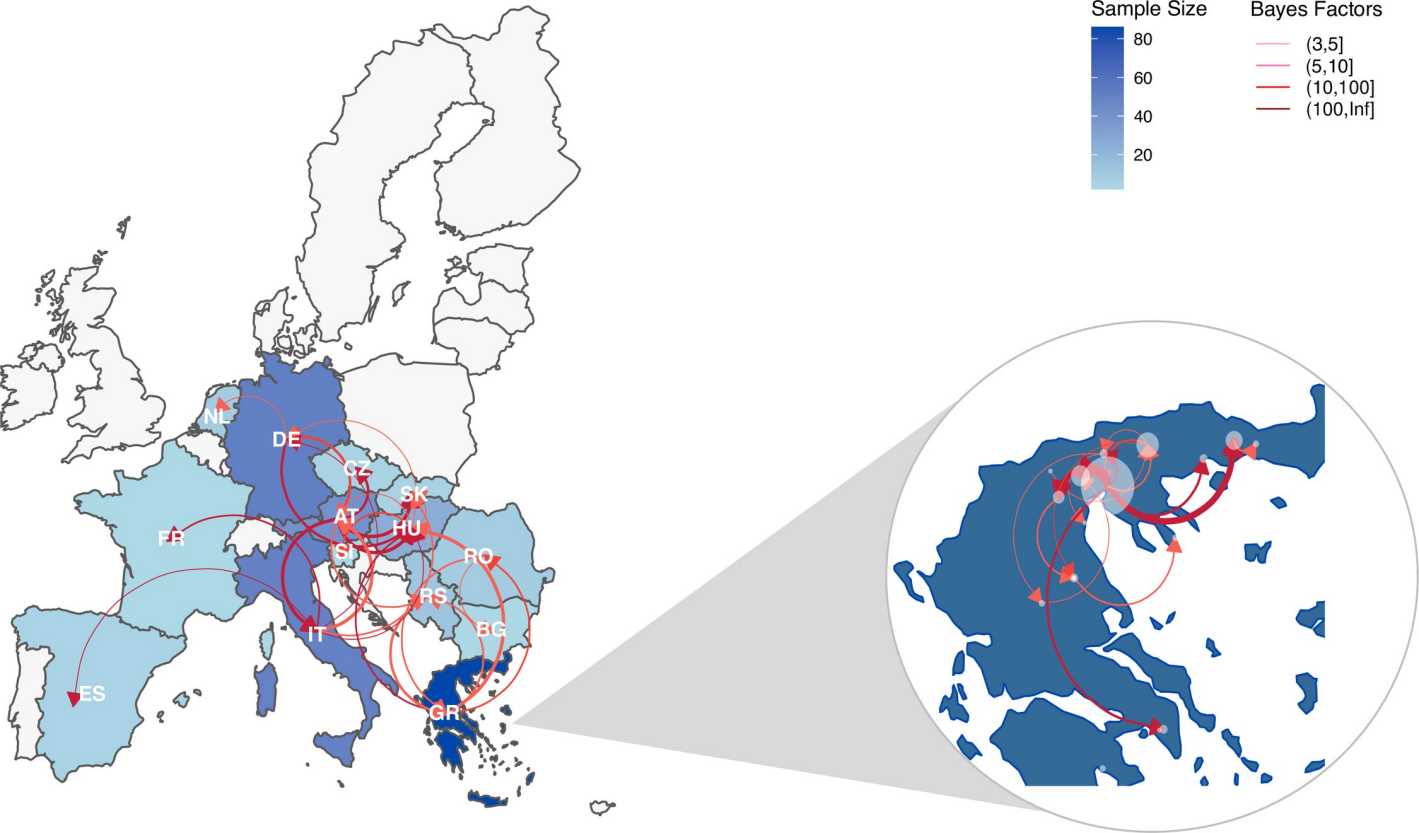
Quantified transmission network of WNV-2a between European countries and within Greece inferred using discrete trait models. The shape of colors on the map indicates the number of samples; the edge weight indicates the median number of transmissions between pairs of countries/regions; the arrow on the edge indicates transmission direction; color of the edge from light to dark indicates Bayes Factor (BF) support from low to high only transmissions with BF>3 are shown). The European shapefile was created using the R package “rnaturalearth” (https://cran.r-project.org/web/packages/rnaturalearth/).

The discrete trait analysis is conducted with the intention of guiding targeted risk surveillance at the country/region level. Here we quantified frequencies of transmission among the 14 European countries where we have available sequences by summarizing the transmission matrix jointly from NS5 and NS3 gene trees. We found transmissions were most likely to occur between neighboring countries. In addition, Austria and Hungary (both in Central Europe) had the most linkages with other countries (Figs 4, S6 and S7A).

We traced the origin and transmission of WNV-2a in the Netherlands and Italy (2019–2020) using newly available genomes in this study. The newly added genomes from the Netherlands were isolated from *Culex pipiens* (common house mosquito), *Gallus gallus domesticus* (chicken), *Passer domesticus* (House sparrow) and *Phylloscopus collybita* (Common chiffchaff) in August–October 2020. According to our phylogeographic analysis, they are genetically closely related to each other, and are most likely transmitted from Germany via a single introduction which occurred between May 2019 and May 2020 (S6 and S7 Figs). Both the 2019 isolates (n = 7, from Treviso, Rovigo, and Padova) and the 2020 isolate (n = 1, from Verona) of WNV-2a human genomes in Italy clustered with isolates from the same areas in Italy in 2016–2018, but more likely had separate origins (S6 and S7 Figs).

Greece contributed the highest number of WNV-2a sequences in Europe, with all belonging to Cluster B. Our analyses suggested WNV has been transmitted from central Europe (Hungary) to Greece and kept circulating within Greece almost annually except in 2015–2016. In 2017, WNV-2a re-emerged in Greece and caused outbreaks in 2018 and continued to circulate during 2019–2021 (S4 and 5 Figs). Between 2017 and 2021, at least six distinct sub-clusters were observed (S6 Fig), three of which were associated with older isolates from Greece dating back to 2013. Sequences from non-neighboring countries (Hungary, Romania, and Bulgaria) were included in the remaining three sub-clusters. Among these, sequences from Hungary collected between 2018 and 2019 were most closely related to sequences from Greece collected within the same time periods (S6 Fig).

**Fig 5:**
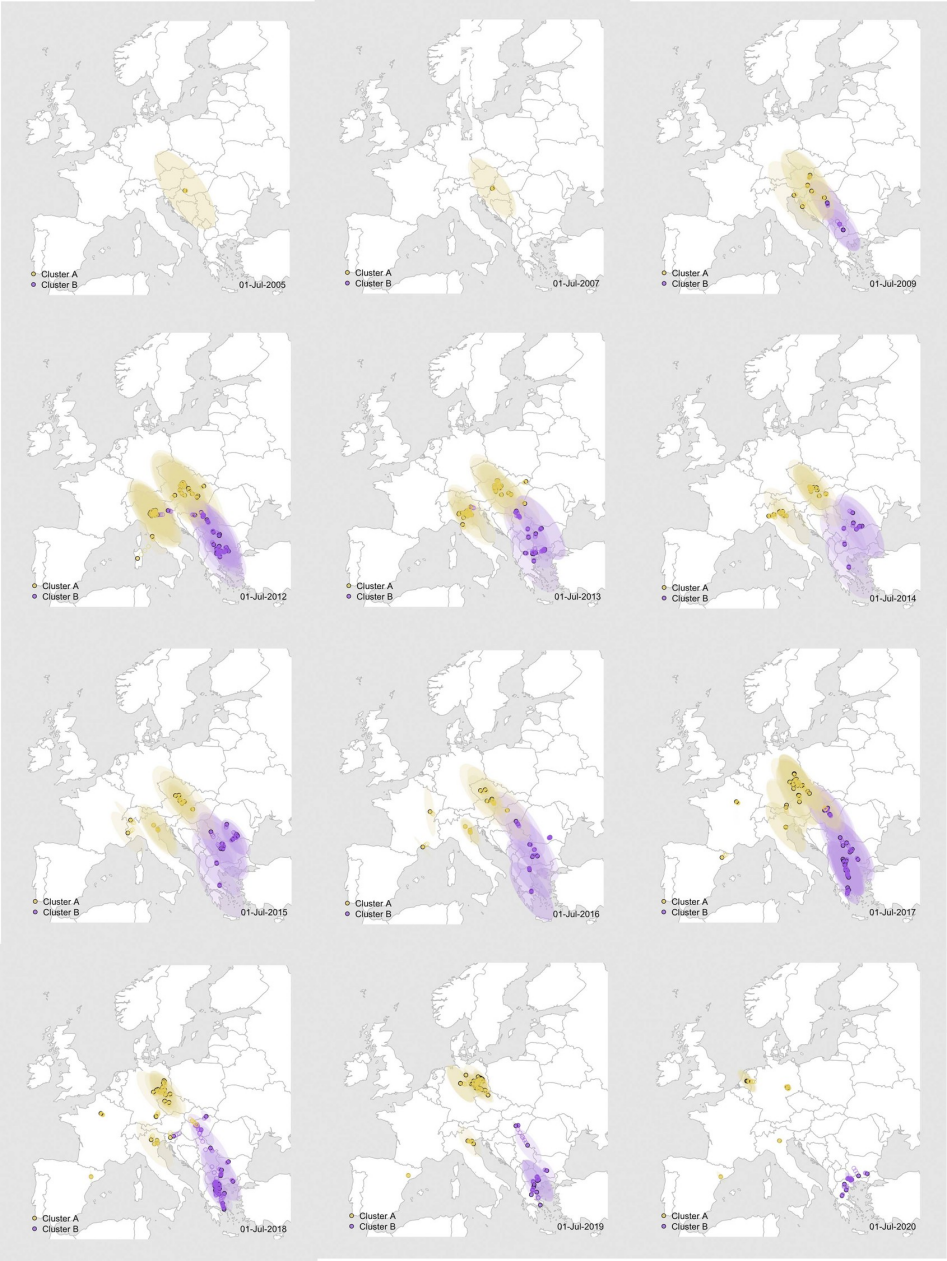
Dispersal history of WNV-2a in Europe between 2004 to 2021. Colors of the dots represent interpolated maximum clade credibility phylogeny positions for clusters A (yellow) and B (purple) from NS3. Please see S1 Movie for the full movie. The European shapefile was created using the R package “maps” (https://cran.r-project.org/web/packages/maps/).

The discrete trait phylogeographic analysis estimated that approximately 19 transmission events between Greece and neighbouring countries occurred in the past decade (Figs 4A and S7A). Countries that had the most frequent transmissions to Greece were Hungary (n = 8), followed by Serbia (n = 5) and Romania (n = 4). The transmissions between Hungary and Greece occurred multiple times between 2010 and 2019, while the transmissions between Greece and other countries occurred mainly in 2012–2013. We further estimated the transmissions within Greece; between-region transmissions mainly occurred in north Greece and spread to the east and south regions (Figs 4B and S7B).

### Continuous dispersal and impact of environmental factors on viral lineage dispersal

By incorporating the coordinates of each sequence isolate, we utilized a continuous phylogeographic model to reconstruct the dispersal history of WNV-2a, aiming to estimate and extract its geospatial spread pathway and further explore the correlation of this dispersal with environmental and ecological factors in a grid format (S1 Text and S1 Movie and Figs 2 and 5). WNV-2a emerged in Central Europe, specifically Hungary, around 2003–2004, and to date, no related genetic sequences have been found in other continents prior to its emergence. One year later, WNV-2a dispersed to the eastern part of Austria and then over the course of 15 years spread to the northwest and western regions for cluster A (including Slovakia, Serbia, Hungary, Austria, Czech Republic, Slovenia, Germany, Netherlands, France, and Spain) and southeastern countries for cluster B (Greece, Romania, Italy, Bulgaria, Serbia, Slovakia, and Slovenia). We could see that WNV emerged and circulated slowly in central Europe at first, then spread to the southeast regions, and then in a westward direction. The frequency and velocity of dispersal was higher in 2013–14, then slowed down, and speeded up again around 2018 (Fig 3F). The periods with high dispersal velocity were consistent with the timing of increased notifications of WNV outbreaks simultaneously in multiple areas as well as the introduction to new geographic regions, e.g., further northwards spread of WNV-2a (Cluster A) towards Germany and the Netherlands. Additionally, the likelihood of dispersal into new locations increases as incidence in the host reservoir rises.

**Fig 6:**
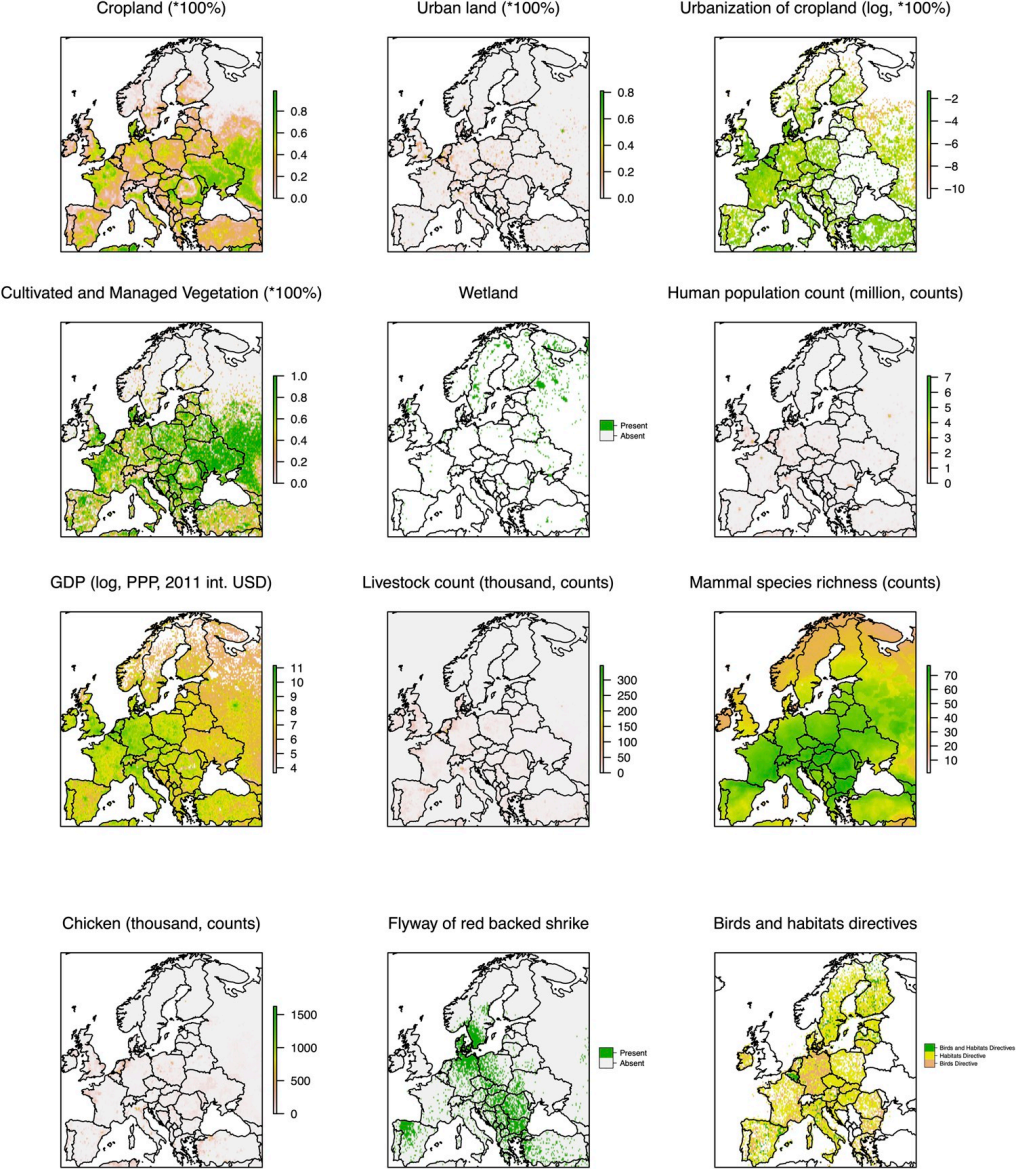
Explanatory factors significantly attract WNV dispersal in Europe. There are eleven factors (out of the total 37 factors being tested) that may attract WNV dispersal with strong statistical support (BF>20, as shown in S1 Table). The first four panels represent the percentage covered by each of the land cover types (Cropland, Urban land, land area changes from cropland to urban land, and Cultivated and Managed Vegetation) in 2015 in each grid cell. The visualizations and full descriptions of all factors are in the (S1 Fig and S2 Table). The unit of each predictor is shown after the predictor name above each panel. The European shapefile was created using the R package “rworldmap” (https://cran.r-project.org/web/packages/rworldmap/).

**Fig 7:**
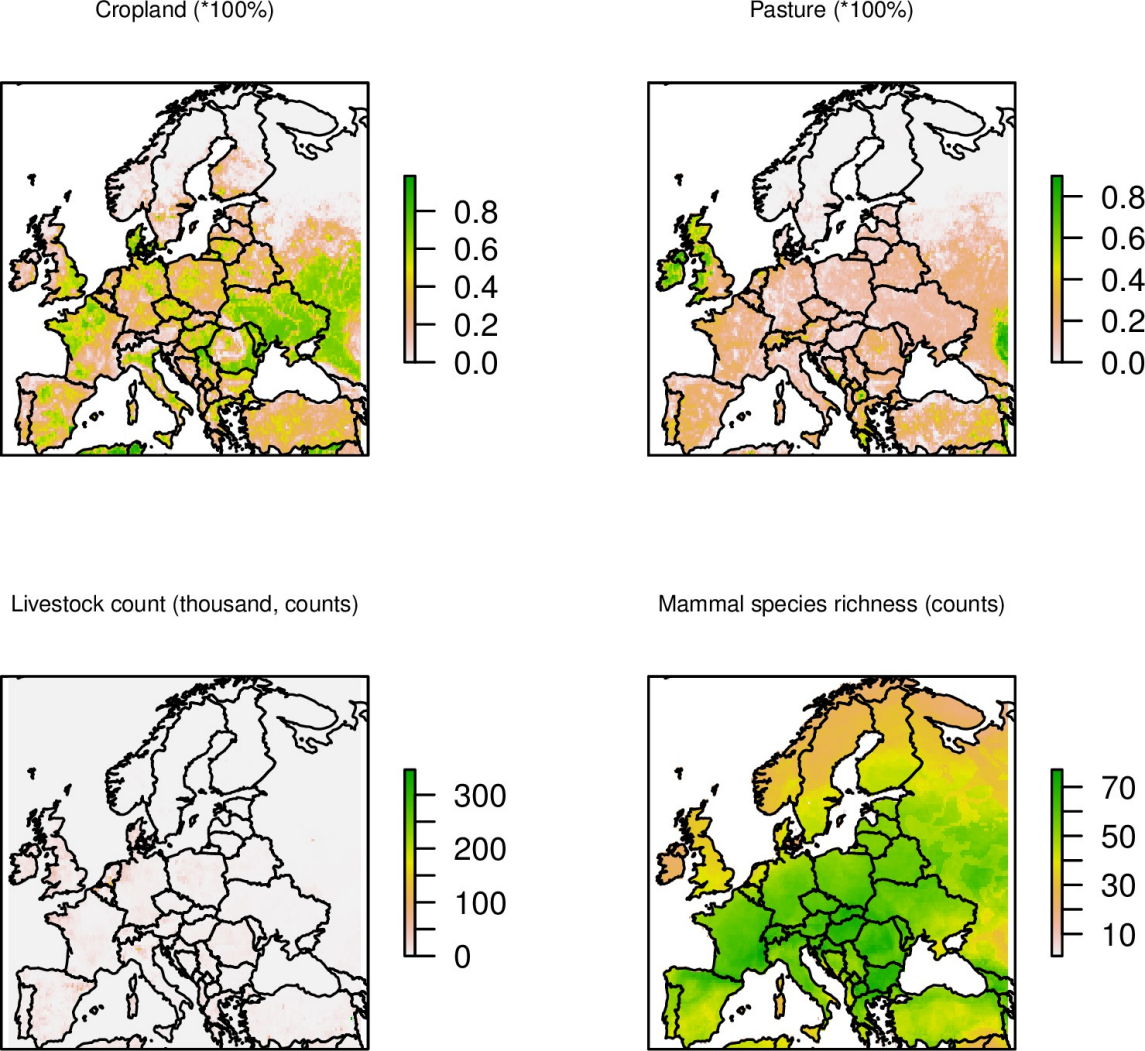
Explanatory factors have a significant impact on WNV dispersal velocity in Europe. There are four factors (out of the total 37 factors being tested) that may speed up WNV dispersal with strong statistical support (BF>20, as shown in S2 Table). The first two panels represent the percentage covered by each of the land cover types (Cropland, Pasture) in 2015 in each grid cell. The visualizations and full descriptions of all factors are in the (S1 Fig and S2 Table). The unit of each predictor is shown after the predictor name above each panel. The European shapefile was created using the R package “rworldmap” (https://cran.r-project.org/web/packages/rworldmap/).

We also extracted the spatio-temporal information embedded in the tree and tested the correlation of the phylodynamic dispersion with gridded predictors (S1 Fig and S2 Table). Through this analysis, we investigated the correlation between the dispersal direction of WNV and variations in the values of environmental variables in the landscape the virus is moving through. Our analysis highlights that WNV-2a had a tendency to be attracted to areas with key drivers indicating high crop and vegetation density, livestock cultivation, and urbanization (Fig 6 and S4 Table). We found that the virus was also likely to be attracted to wetlands, protected bird and habitat areas, and migratory bird flyways (Fig 6 and S4 Table). In addition to the directionality of spread, we observed that factors related to high farming density (defined by high crop and vegetation density and livestock cultivation) may accelerate WNV dispersal velocity in Europe. The reported conductance factors are significantly supported by employing a least-cost model on four different datasets (NS3 complete, NS3 cluster A, NS3 cluster B and NS5 complete), demonstrating a positive regression coefficient with a Bayes Factor exceeding 20. Additionally, the associated Q statistic consistently exhibits a positive value of over 90% (Fig 7 and S5 Table and S1 Data). We validated our findings using the CIRCUITSCAPE (CS) model, and the results were broadly consistent with those obtained through the least-cost model, on the NS3 complete dataset. Note that the CS model is significantly more computationally demanding than the least-cost model [36], and both models have consistently identified the same top four drivers (including cropland, pastureland, livestock counts, and mammal richness). These findings expand our understanding of the ecological drivers of WNV spread, by adding the important association with factors related to land use.

### Impact of climate/bio-diversity changes on WNV population genetic diversity via time

As the dynamics of host populations (mosquitoes and birds) vary widely in different months or years, it is important to understand how these variables could further affect WNV genetic diversity or population dynamics. Therefore, we examined the relationship between the temporal change of the effective population of WNV-2a lineage and climate change coefficients in a log-linear regression model. In this study, the covariates of interest included the time-varying climate-related factors (including air temperature, precipitation, wind speed and direction, and leaf area index) and the factors related to the trend of population changes in different types of birds in Europe (S2 Fig and Table 1).

**Table 1:** Time-varied factors with significant impacts on effective population size via time*.

Covariates	Time unit	Coefficient mean	95% BCI lower	95% BCI upper
Surface air temperature at 2m	monthly	0.18	0.08	1.68
Precipitation	monthly	-1.17	-1.89	-0.47
Leaf area index, low vegetation	monthly	1.22	-0.62	3.11
Leaf area index, high vegetation	monthly	1.15	-0.81	3.04
10m u-component of wind (eastward)	monthly	-0.70	-1.29	-0.07
100m u-component of wind (eastward)	monthly	-0.76	-1.74	-0.12
10m v-component of wind (northward)	monthly	-0.75	-1.51	0.08
100m v-component of wind (northward)	monthly	-0.60	-1.75	0.68
10m u-component of neutral wind (eastward)	monthly	-1.00	-1.72	-0.24
10m v-component of neutral wind (northward)	monthly	-0.56	-1.65	0.64
10m wind speed	monthly	-1.11	-1.85	-0. 40
Instantaneous 10m wind	monthly	0.93	-0.22	1.66
Bird index-farmland birds	yearly	-0.50	-1.13	-0.12
Bird index-forest birds	yearly	-0.16	-2.06	1.83
Bird index-other birds	yearly	-0.31	-0.76	0.16
Bird index-*Galliformes*	yearly	0.00	-0.14	0.13
Bird index-*Passeriformes*	yearly	-0.29	-0.88	0.25
Bird index-*Strigiformes*	yearly	-0.04	-0.16	0.08
Bird index-*Accipitriformes*	yearly	-0.03	-0.08	0.01
Bird index-*Galliformes*	yearly	-0.03	-0.20	0.19
Bird index-*Coliformes*	yearly	-0.09	-0.40	0.24
Bird index-*Falconiformes*	yearly	-0.19	-2.22	1.65

*The correlation between temporal factors (n = 22) and the log effective population size (Ne) of WNV-2a over time. The 95% Bayesian credibility interval (BCI) of covariate effect size lying either entirely above or below zero indicates a statistically significant association. Positive correlations are shown in red, and negative correlations are depicted in green.

We found a significant positive association between the population history of WNV-2a and changes in air temperature in Europe between 2004 and 2021. This concordance was supported by a posterior mean covariate effect size of 0.18 (95% Bayesian credibility interval (BCI) of (0.08, 1.68) (Table 1). In contrast, wind speed in the eastward direction (rather than the northward direction) at a height of 10 and 100 meters above the surface of the Earth, monthly total precipitation, and the trends of farmland bird populations (the change in the relative abundance of 39 common bird species which are dependent on farmland for feeding and nesting and are not able to thrive in other habitats) were negatively correlated. In comparison, northward wind speed, the leaf area index of different types of vegetation and changes of bird index in other categories did not have a significant impact on the genetic diversity changes of WNV-2a viruses, with 95% BCI of covariate effect size containing zero (Table 1).

## Discussion

In this study, we used spatially explicit phylogeographic and phylodynamic inference to reconstruct the dispersal patterns and dynamics of WNV strains dominant in Europe in the past twenty years. Although the evolution of WNV has previously been examined in individual European countries in different time spans [16–18], this study has employed much more comprehensive and sophisticated phylodynamic models to incorporate sequence data, ecological data and epidemiology data, and quantified the evolution and dispersal history across Europe based on sequence data and metadata which has been updated by our European consortium up to 2021. In line with earlier research, we also observed distinct evolutionary histories for lineages WNV-1 and WNV-2, as well as for sub-lineages WNV-2a and WNV-2b within the overall spread of WNV. We further enhanced our analysis by quantifying separate complex dynamics using comparative analyses of lineage dispersal statistics. We additionally assessed the potential impact of predictors that influence WNV dispersal and diversity in Europe to gain an improved understanding of risk.

The current predominant sub-lineage WNV-2a has only been found within Europe but we cannot rule out the possibilities of cross-continent introduction due to under-sampling in non-European regions. One hypothesis is that the ancestor of WNV-2a was initially introduced to Europe (presumably coming from Africa [19]) via long-distance migratory birds, and after spreading to and circulating in local bird populations and other hosts, the virus evolved, diversified, and spread throughout the European continent. WNV-2a has diverged into 2 clusters (Cluster A and B) and spread to the west (Cluster A) and to the south (Cluster B). We highlighted that areas with important levels of agricultural activities may accelerate WNV dispersal velocity as well as attract the spread direction of WNV in Europe. Agricultural activities cause a dramatic degradation of natural ecosystems and loss of mosquito and bird diversity, but increase the availability of aquatic habitats, all increasing the risk of vector-borne pathogen transmission [36]. This is supported by the observation of the correlation between WNV circulation and the decline in farmland bird species in Europe in the past two decades, suggesting the invasion of WNV might have a preferential impact on the population of common bird species that are dependent on farmland for feeding and nesting. Meanwhile, the loss of habitat may force birds to change migration routes, increasing the possibility of WNV spreading to new territories [37]. In addition, we showed that WNV is likely to spread to areas with a high degree of urbanization, where the ecological imbalance caused by anthropogenic changes is even worse, characterized by less balanced mosquito communities and higher abundance of *Culex pipiens* (S5D Fig), which prefers urban environments due to the availability of artificial aquatic habitats, the reduced number of predators and the presence of warmer environment temperature [38]. It is also worth noting that *Culex* hybrids from the *pipiens* and *molestus* forms have been found in Europe, which can serve as a bridge for WNV between birds and humans, and the proportions of hybrids are differentially affected by temperature, e.g., 17% of *Culex* are hybrids in Greece, and the figure is 6–15% in the Netherlands [39–42]. However, the association between the presence of different mosquito species and the distinct dynamic histories of WNV variants (WNV-2a and WNV-2b) remains unclear and requires further investigation.

While the presence of agricultural activity seemed most strongly associated with northward dispersal of WNV, for central Europe we found a strong relationship between the presence and movement of birds and WNV spread. This is further verified by the finding that WNV was more likely to spread towards areas with the presence of wild bird species and habitats covered in the bird directive policy (ECDC). In fact, almost all outbreaks in Greece, Romania, Italy and other European countries started near wetlands and subsequently areas with high numbers of migratory birds [43–46]. Our research indicated that WNV may be "attracted" to areas with high bird habitat density and to the flyway of *Passeriformes* (S5C Fig), a large bird order that includes migratory species such as the Red-backed Shrike (*Lanius collurio*) [20,47–49]. Consistently, we found WNV-2a in Europe spread at a comparably high dispersal velocity (88km/yr to 218km/yr) and, therefore, is more likely correlated to bird movement than the travel of *Culex* mosquitoes (approx. 500m to 2km/yr) [50,51]. Our findings suggest that while both infected birds and mosquitoes play a role in the risk of WNV transmission, bird movements can introduce the virus into new regions and infect mosquitoes far away. However, it seems the estimated dispersal velocity and geographic expansion of WNV are limited by the species of susceptible birds. In contrast, long-migratory wild birds mainly belonging to *Anseriformes* have contributed to a much greater geographic expansion of highly pathogenic avian influenza virus H5Nx of 2.3.4.4b clade, with a rate of 3000–5000 km/yr [52]—approximately ten times higher than that of WNV-2a. The viral genomes from birds may not fully represent all species that serve as WNV amplifying hosts, and further investigation into a wider range of bird species will be necessary to identify key amplifying hosts for WNV.

Future surveillance should include more sustained and collaborative efforts to fill in the gaps in WNV genomic sampling throughout Europe. Since the early 2000s, WNV circulation has been continuously monitored in some European countries with varying numbers of human and equine cases. Surveillance of mosquitoes and birds has proven to be useful for early detection of WNV circulation and identification of enzootic areas [21,53–56]. The available data do reflect enhanced efforts in some countries. In Greece, active mosquito surveillance, especially in the Central Macedonia Region, has been performed since 2010 when the virus was first identified in the country [21,53,57]. In Italy, a multi-species national surveillance plan was implemented in 2002, following the first WNV outbreak [15]. Since 2020, the national surveillance plan has been enhanced and fully integrated into a “One Health” approach, including human, wild bird, equine, and mosquito surveillance for the early detection of WNV activity [58]. In the Netherlands, the number of screened samples has steadily increased each year since 2016, with a rise from 952 samples in 2016 to 7030 samples in 2021. However, despite this increase in screening, no WNV sequence was detected during this period, with the exception of 2020 [59]. Our phylogeographic analysis suggests that this detection in 2020 was likely due to transmission from Germany. In Germany, a nationwide wild bird surveillance network for zoonotic arboviruses was established more than ten years, following the first Usutu virus outbreak in this country [60], and has recently been extended to zoo birds. By this well-functioning network with dead and live bird surveillance, it was possible to detect the first entry of WNV in 2018, and to follow the annually spreading tendency from the endemic area in East Germany [16,61]. On the contrary, the number of publicly accessible whole genomes WNV genomes is insufficient to reconstruct the complete phylogeographic spread history, e.g., from Central and Southeast Europe (such as Croatia [62], Bulgaria [54], Slovenia [63], and Turkey [64,65]) as well as Southwest Europe (France, Spain [66] and Portugal [67]) where WNV have been detected in both humans and animals [2] (S3 Fig).

In addition to the factors discussed above, climate changes over the past two decades have been found to influence changes in viral genetic diversity over time. We found that higher temperature is correlated with high WNV genetic diversity during the entire history of WNV-2a spread in Europe, which is in line with previous findings [68,69]. High temperatures may stimulate the geographical expansion of suitable arbovirus regions [70]. Other epidemiological modelling studies have found that mild winters and drought have been associated with WNV disease outbreaks [70,71]. We also found that precipitation and wind speed had negative correlations, which could be explained by WNV infections mostly occurring during the mosquito season in summer [1] and also the dry season in most European countries. In addition, we found that the direction of wind also matters. The underlying mechanism remains to be further investigated, though one study found that wind can strongly reduce mosquito catches so that could be a practical means of protecting humans or animals from mosquitoes [72].

One important aspect of our study was to evaluate the quality of the datasets used for predicting the spread of WNV and found that the data sources were generally of high quality, with good accuracy, reliability, and availability. However, we did identify one potential predictor, the distribution of *Culex pipiens*, as having lower quality due to the lack of completeness in geographical coverage. Future work could use, for example, outputs from Mosquito Alert (http://www.mosquitoalert.com/en/access-to-mosquito-alert-data-portal/), which has started collating reports of *Culex* sightings through citizen science since 2020. Similarly, forest-related species richness and livestock density were of lower quality; livestock density was a significant factor for WNV dispersal highlighting the need for these data sources to be regularly updated and ensured for accuracy.

In order to maximize the sample size and geographic coverage while reconstructing the phylogeographic spread history of WNV in Europe over the past 20 years, we opted to use the NS3 gene dataset in the continuous model, particularly for sequences isolated around the 2010s in central and southern Europe. Although WGS would give improved phylogenetic resolution, the NS3 gene has sufficient variation for time-scaled phylogenetic tree inference. As more WGS data becomes available in Europe, future studies focusing on WNV spread in more recent times will be performed.

The risks of zoonotic diseases and their spread increase with globalisation, climate change and changes in human behaviour, giving pathogens numerous opportunities to colonise new territories and evolve into new forms. We used virus genomics to investigate the emergence of WNV in Europe, as well as its rapid spread, evolution in a new environment and establishment of endemic transmission. Most importantly, our study suggested targeted regions in central Europe need further investigation, including areas where WNV-2a was more likely to cluster (with a high percentage of urban land and farming activities, high coverage of wetlands, as well as areas with the presence and movements of migrating and resident birds) and areas with higher farming density tended to accelerate the dispersal of WNV.

### Ethics approval

An approval by an ethics committee was not applicable.

## Supporting information

S1 Movie Phylogeny-inferred dispersal history of WNV-2a in Europe between 2004 to 2021. Colors of the dots represent interpolated maximum clade credibility phylogeny positions for clusters A (yellow) and B (purple) from NS3. The base map of the movie is displayed using R package “maps” (https://cran.r-project.org/web/packages/maps/). This world map (within package maps, updated in 2013) is imported from the public domain Natural Earth project (the 1:50m resolution version), The Natural Earth data set is in the public domain and available from https://www.naturalearthdata.com. The data from the time-scaled phylogenetic MCC tree is overlayed using the author’s own custom R code which makes use of R package ape (https://cran.r-project.org/web/packages/ape/index.html).
(GIF)

S1 Text Supplementary Materials and Methods. (DOCX)

S1 Table Sequences and metadata of WNV lineage 2a used in this study. (DOCX)

S2 Table Predictors of West Nile Virus Dispersal in Europe Assessed for Quality. (DOCX)

S3 Table Predictors for viral genetic diversity over time. (DOCX)

S4 Table Results of drivers on viral dispersal direction*. (DOCX)

S5 Table Results of drivers on viral dispersal velocity*. (DOCX)

S1 Data Full results of impact on velocity using LC and CS models. (XLSX)

S1 Fig Distribution of predictors for dispersal of West Nile virus in Europe. The Definition for each predictor is shown in the (S2 Table). Data were log-transformed where necessary for better visualization. The unit of each predictor is shown after the predictor name above each panel. The European shapefile was created using the R package “rworldmap” (https://cran.r-project.org/web/packages/rworldmap/).
(DOCX)

S2 Fig Distribution of predictors for viral genetic diversity over time. Data in four months (January, April, July, and October) were shown for each predictor. Definition for each predictor is shown in the (S3 Table). The unit of each predictor is shown after the predictor name above each panel. The European shapefile was created using the R package “rworldmap” (https://cran.r-project.org/web/packages/rworldmap/).
(DOCX)

S3 Fig Surveillance and sequencing effort of WNV in Europe. (a) Comparison between cumulative human cases reported by ECDC (between 2008–2021, total n = 4188) and the number of WNV sequences (between 1971–2021, total n = 485) isolated from 22 different countries. (b) The sequencing effort (ratio of the number of sequences available to the number of human cases reported) per country is shown on the map: red from light to dark indicates the ratio from low to high; green indicates no sequence available although human cases have been reported; grey indicated neither human cases nor sequences are available. The European shapefile was created using the R package “raster” (https://cran.r-project.org/web/packages/raster/).
(DOCX)

S4 Fig Number of sequences per lineage in different countries and years. (DOCX)

S5 Fig Distributions of WNV-2a sequences from different hosts. (a) Sequences from different hosts on maps. The colors for hosts are consistent in a-d. (b) Relative frequencies of sequences sampled from 5 different hosts. (c) Relative frequencies of sequences sampled from different bird orders. (d) Relative frequencies of sequences sampled from different mosquito species. The European shapefile was created using the R package “maps” (https://cran.r-project.org/web/packages/maps/).
(DOCX)

S6 Fig Time-scaled MCC trees of WNV mapping with countries. Trees were reconstructed using sequences of ns3 gene (a) and ns5 gene (b) mapping with countries (n = 14).
(DOCX)

S7 Fig Quantified WNV transmission networks. Transmission networks inferred from the joint analysis of ns3 and ns5 phylogenies of WNV-2a sequences. (a) between European countries. (b) between regions in Greece. The size of node indicates the number of samples; edge weight indicates the median number of transmissions between pairs of locations; the arrow on edge indicates transmission direction; color of the edge from light to dark indicates Bayes Factor (BF) support from low to high only transmissions with BF >5 are shown). The correlated farms are grouped together. Nodes with no link to the others indicated no significant transmissions with other areas although sequences have been sampled.
(DOCX)

S8 Fig Geographic distributions of WNV-2a sequences dataset. a) ns3 gene, b) ns5 gene, c) full genomes and d) the number of sequences for each dataset. The European shapefile was created using the R package “maps” (https://cran.r-project.org/web/packages/maps/).
(DOCX)
