# Supplementary figures and images for: West Nile virus spread in Europe: Phylogeographic pattern analysis and key drivers
Source: PLoS Pathog. 2024 Jan 25;20(1):e1011880. doi: 10.1371/journal.ppat.1011880 (PMC10810478; doi:10.1371/journal.ppat.1011880)

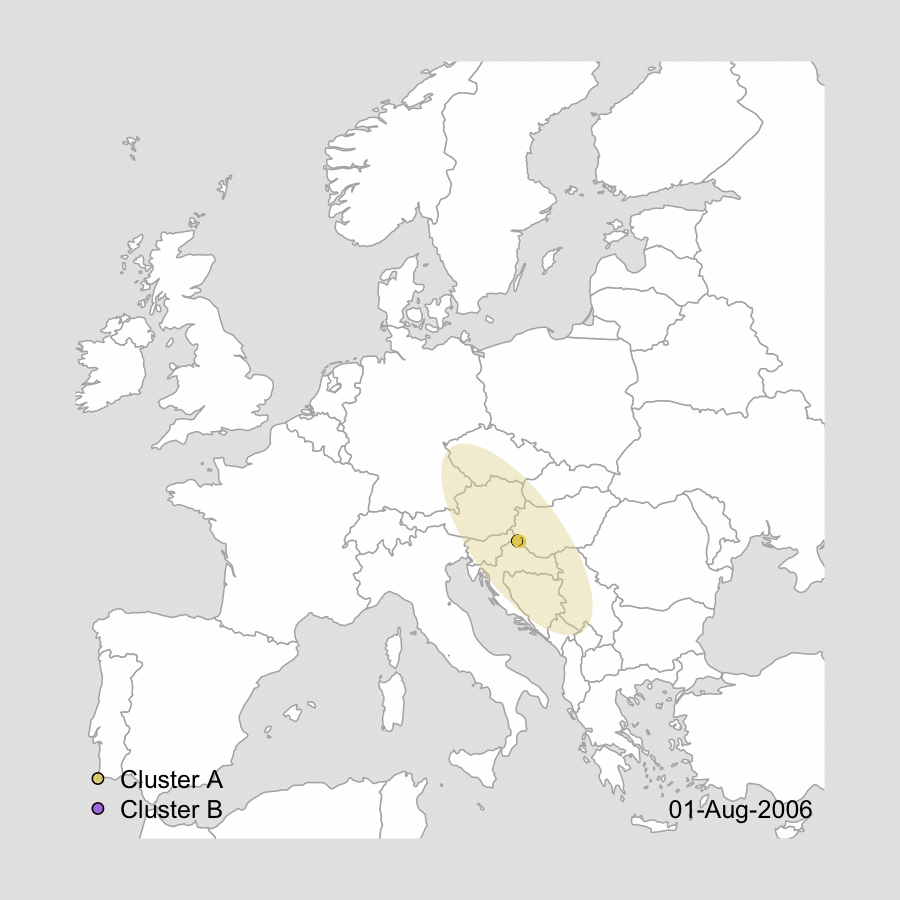

Supplement: S1 Movie — Colors of the dots represent interpolated maximum clade credibility phylogeny positions for clusters A (yellow) and B (purple) from NS3. The base map of the movie is displayed using R package “maps” (https://cran.r-project.org/web/packages/maps/). This world map (within package maps, updated in 2013) is imported from the public domain Natural Earth project (the 1:50m resolution version), The Natural Earth data set is in the public domain and available from https://www.naturalearthdata.com. The data from the time-scaled phylogenetic MCC tree is overlayed using the author’s own custom R code which makes use of R package ape (https://cran.r-project.org/web/packages/ape/index.html). (GIF) [file ppat.1011880.s001.gif]
