# Supplementary material for: West Nile virus spread in Europe: Phylogeographic pattern analysis and key drivers
Source: PLoS Pathog. 2024 Jan 25;20(1):e1011880. doi: 10.1371/journal.ppat.1011880 (PMC10810478; doi:10.1371/journal.ppat.1011880)
Supplement: S1 Text — (DOCX) [file ppat.1011880.s002.docx]

# S1 Text Supplementary Materials and Methods

## Genome Sequence data collection

We include new previously unpublished WNV-2 genomes collected from Italy (n=8) and the Netherlands (n=6) between 2019 and 2020 in this study. Virus isolation, identification and sequencing are as previously described^1-3^. Sequences have been submitted to GenBank with accession IDs OP561452-OP561459, OP762592-OP762597 (S1 Table).

Apart from the new WNV genomes obtained in this study, we downloaded all available nucleotide sequences of WNV isolated from Europe as of 02 June 2022 from NCBI ([www.ncbi.nlm.nih.gov](http://www.ncbi.nlm.nih.gov)). To identify possible cross-continent transmission, we blasted our European WNV dataset against sequences database at NCBI using Geneious Prime 2021.1.1 (<https://www.geneious.com>), the sequences from Europe within the same lineages are distinct from those isolated in other continents. We counted in total 485 WNV sequences (from unique samples), including 226 full genomes sequences over a 50-year span from 1971 to 2021. Metadata of WNV sequences that could not be publicly released were updated by the aforementioned European collaborative consortium. For example, we have adjusted travel history of human cases (if known) to locate the original source of infection. The coordinates of samples were deployed in Figshare (<https://doi.org/10.6084/m9.figshare.21444783>).

## Phylodynamic reconstructions

Sequences were aligned with MAFFT^4^. Phylogenetic trees were first generated using IQtree^5^ employing maximum likelihood (ML) under 1000 bootstraps. Sequences with >5% ambiguous nucleotide sites were excluded and for sequences 100% identical to one another, only one of the sequences was included. The nucleotide substitution model used for all phylogenetic analyses was HKY with a Gamma rate heterogeneity among sites with four rate categories. The temporal qualities of the sequence data were measured with TempEst v1.5^6^.

We further reconstructed time-scaled phylogenies of WNV-2a, which is the predominant lineage found in Europe, including all WNV-2a whole genome sequences (n=208), NS3 gene sequences (n=275), and the NS5 gene sequences (n=232), separately (S1 Table). The NS3 gene dataset has the largest amount of sequences and covers the widest range of geographic areas (S1 Table and S8 Fig). Therefore, we mainly reported the results of the continuous phylogeographic analysis using NS3 dataset in the main text. We first tested the temporal signals of the ML trees generated using WNV-2a full genomes (R=0.62) and compared these results with analysis of the trees generated using NS3 (R=0.17) and NS5 gene (R=0.25), respectively. We specified a strong prior for the evolutionary clock rate of WNV (both NS3 and NS5 gene dataset) with a lognormal distribution and a mean of 4x10^-4^ subst/site/year with Standard Deviation of 5x10^-4 7^. Phylodynamic analyses using WNV-2a sequences were conducted using time-scaled Bayesian phylogenetic methods in BEAST version 1.10.4^8^. The best fitted models were determined using stepping-stone sampling^9^, which resulted in the selection of HKY+Gamma+4 substitution model—an uncorrelated relaxed molecular clock model which assumes each branch has its own independent rate^10^ and Skygrid^11^ coalescent model. For each analysis, the Monte Carlo Markov Chain (MCMC) was run for 10^8^ steps and sampled every 10^4^ steps.

We further estimated the transmissions between countries and within countries by using linked parameters options in BEAST ^8^ to jointly estimate the between country transmissions using both the NS5 gene sequences and NS3 gene sequences (which have different phylogenies). We used an asymmetric model and incorporated the Bayesian Stochastic Search Variable Selection procedure (BSSVS) to identify a sparse set of transmission rates that identify the statistically supported connectivity^12^. We also estimated the expected number of transmissions (jumps) between countries and within countries using Markov rewards^13^. In addition, a continuous phylogenetic diffusion model with a Relaxed Random Walk extension^14^ was further applied to explore the geographical spread of WNV in continuous space.

## Spatial and temporal predictors of WNV dispersal

### **Collection of predictor data indicating drivers of disease emergence and/or spread**

We collected a list of potential predictors that are thought to be associated with viral dispersal direction & velocity (n=37) as well as time-varied predictors that may correlate with viral genetic diversity variations over time (n=22). Data sources, original resolutions, along with the definitions are provided in the S2 Table and S3 Table.

Predictors thought to be associated with viral dispersal direction & velocity (n=37) were mainly assigned into five categories, including 1) climate and weather, 2) land use and cover, 3) topography, 4) socio-economic factors and 5) biodiversity (S2 Table). The predictors used in this study were adapted from our previous analysis of the drivers of discovery of human infective RNA viruses^31^. We also included new predictors specifically relevant for arboviruses (e.g., wetland concentration, occurrence status of *Culex pipiens*) (S1 Fig). Resolution of predictors ranged from 30" to regional level, and all data were rescaled to 0.25° (~28km) where possible. Collection of data on spatial and temporal drivers of WNV dispersal and the related modelling process were performed using the R version 3.6.3 (R Foundation for Statistical Computing, Vienna, Austria, 2020). For each of the potential predictors, we assessed the quality of the data sources used in terms of their ability to represent the underlying driver as described by Horigan, V et al^15^. Specifically, we assessed each driver against six characteristics (Accuracy & Precision, Reliability & Consistency, Timeliness, Completeness, and Availability & Accessibility, Granularity). Overall, the data sources were of very good quality, scoring well for accuracy, reliability and availability, including the factors regarding land use which the model output as influential on WNV spread. The data for *Culex pipiens* distribution was assessed to have the lowest quality amongst the factors included, due to the lack of completeness in geographical coverage (S2 Table).

The temporal factors (n=22) we tested included two parts: one group was climate related data obtained from ERA5 with a spatial resolution of 0.25° (<https://www.ecmwf.int/en/forecasts/datasets/reanalysis-datasets/era5>) including monthly values of 2m air temperature, total precipitation, northward wind speed (100m, 10m neutral, and 10m); eastward wind speed (100m, 10m neutral, and 1), the leaf area index of high vegetation, as well as the leaf area index of low vegetation (S3 Table). We also obtained the common bird population index in Europe between 2004 to 2019 from The Pan-European Common Bird Monitoring Scheme (PECBMS) (<https://pecbms.info/trends-and-indicators/species-trends/>), and further grouped the data into 3 types (farmland, forest and others) and bird orders (extract bird species belong to 7 bird orders that matched with the bird types in our WNV sequence data: *Accipitriformes, Charadriiformes, Coliformes, Falconiformes, Galliformes, Strigiformes and Passeriformes*) independently (S5C Fig).

### **Prediction of drivers on viral dispersal direction**

We tested the associations between the above set of potential predictors (S2 Table) of presence and dispersal of WNV using R package “seraphim”^16^, which has been developed to study the time-scaled phylogeography in an environmental context; it extracts the spatio-temporal information from phylogenetic trees and uses this information to calculate and plot dispersion statistics.

We first tested the association between the dispersal directions for each branch of WNV in the phylogenetic tree (from an ancestral node to its descendants), with the equivalent paths corresponding to trajectories through the predictor maps from the ancestral node locations to the descendant locations. We tested if the virus tended to remain in areas with lower/higher environmental values, and/or the tendency of the lineages to disperse towards lower/higher values of the predictive factors, by estimating the Bayes factor (BF) comparing values explored under the inferred model with the null dispersal model simulated along the tree ^16^. We considered a BF value >20 as strong support for a significant correlation between the factors and dispersal directions.

### **Prediction of drivers on viral dispersal velocity**

We also tested the impact on diffusion rate (or dispersal duration) by examining the association between dispersal durations and environmental distances computed for each branch of the tree. on We estimated the value Q which measures the correlation between phylogenetic branch durations and environmental distances, for the phylogenies generated using four datasets separately (NS3 full, NS5 full, NS3 A cluster, and NS3 B cluster), by using the “least-cost” path model, which uses a least-cost algorithm to determine the route taken between the start and end locations of phylogenetic branch within the predictor raster cells^17^. Following the methodology described by Dellicour et al^17^, for each of the environmental factors, we generated three scaled rasters by transforming the original raster cell values with the following formula: vt=1 + k(vo/vmax), where vt and vo are the transformed and original cell values, respectively, and vmax is the maximum cell value recorded in the raster. Here k (k=10, 100 and 1000) is a rescaling parameter that tests different strengths of raster cells relative to the conductance (positive correlation) or resistance (negative correlation), with a minimum value set to “1”. Factors with a positive regression coefficient and BF greater than 20, and a positive value for greater than 90% Q statistics are regarded to have a substantial positive association with dispersal velocity of virus lineages. Furthermore, we validated the results using the CIRCUITSCAPE (CS) model, a random walk dispersal model based on circuit theory ^18^.

### **Drivers on changes of viral population diversity over time**

We applied the skygrid-GLM model^19^ to jointly infer the WNV effective population size along with the coefficient that relates it to the time-varied predictors (n=22) (S3 Table, S5C Fig). Here we examine the temporal relationship between the demographic history of WNV and the data describing climate-, land-use- and bio-diversity changes in Europe between 2004 and 2021 (defined by analysis incorporating the datasets described in the S3 Table and S2 Fig).

# Reference

1 Rappole, J. H. & Hubalek, Z. Migratory birds and West Nile virus. *J Appl Microbiol* **94 Suppl**, 47S-58S (2003). https://doi.org:10.1046/j.1365-2672.94.s1.6.x

2 Tsioka, K. *et al.* Detection and molecular characterization of West Nile virus in Culex pipiens mosquitoes in Central Macedonia, Greece, 2019-2021. *Acta Trop* **230**, 106391 (2022). https://doi.org:10.1016/j.actatropica.2022.106391

3 Barzon, L. *et al.* Early start of seasonal transmission and co-circulation of West Nile virus lineage 2 and a newly introduced lineage 1 strain, northern Italy, June 2022. *Euro Surveill* **27** (2022). https://doi.org:10.2807/1560-7917.ES.2022.27.29.2200548

4 Katoh, K. & Standley, D. M. MAFFT: iterative refinement and additional methods. *Methods Mol Biol* **1079**, 131-146 (2014). https://doi.org:10.1007/978-1-62703-646-7_8

5 Minh, B. Q. *et al.* IQ-TREE 2: New Models and Efficient Methods for Phylogenetic Inference in the Genomic Era. *Mol Biol Evol* **37**, 1530-1534 (2020). https://doi.org:10.1093/molbev/msaa015

6 Rambaut, A., Lam, T. T., Carvalho, L. M. & Pybus, O. G. Exploring the temporal structure of heterochronous sequences using TempEst (formerly Path-O-Gen). *Virus Evol* **2** (2016). https://doi.org:ARTN vew00710.1093/ve/vew007

7 Hadfield, J. *et al.* Twenty years of West Nile virus spread and evolution in the Americas visualized by Nextstrain. *Plos Pathog* **15**, e1008042 (2019). https://doi.org:10.1371/journal.ppat.1008042

8 Suchard, M. A. *et al.* Bayesian phylogenetic and phylodynamic data integration using BEAST 1.10. *Virus Evol* **4** (2018). https://doi.org:10.1093/ve/vey016

9 Baele, G. *et al.* Improving the Accuracy of Demographic and Molecular Clock Model Comparison While Accommodating Phylogenetic Uncertainty. *Mol Biol Evol* **29**, 2157-2167 (2012). https://doi.org:10.1093/molbev/mss084

10 Drummond, A. J., Ho, S. Y., Phillips, M. J. & Rambaut, A. Relaxed phylogenetics and dating with confidence. *PLoS Biol* **4**, e88 (2006). https://doi.org:10.1371/journal.pbio.0040088

11 Hill, V. & Baele, G. Bayesian estimation of past population dynamics in BEAST 1.10 using the Skygrid coalescent model. *Mol Biol Evol* (2019). https://doi.org:10.1093/molbev/msz172

12 Lemey, P., Rambaut, A., Drummond, A. J. & Suchard, M. A. Bayesian phylogeography finds its roots. *PLoS Comput Biol* **5**, e1000520 (2009). https://doi.org:10.1371/journal.pcbi.1000520

13 O'Brien, J. D., Minin, V. N. & Suchard, M. A. Learning to count: robust estimates for labeled distances between molecular sequences. *Mol Biol Evol* **26**, 801-814 (2009). https://doi.org:msp003 [pii]10.1093/molbev/msp003

14 Lemey, P., Rambaut, A., Welch, J. J. & Suchard, M. A. Phylogeography takes a relaxed random walk in continuous space and time. *Mol Biol Evol* **27**, 1877-1885 (2010). https://doi.org:10.1093/molbev/msq067

15 Horigan, V., Kelly, L., Papa, A., Koopmans, M.P.G., Sikkema, R.S., Koren, L.G.H., Snary, E.L. . Assessment of data quality for drivers of disease emergence. *WOAH Rev Sci Tech* **41** (2023).

16 Dellicour, S., Rose, R., Faria, N. R., Lemey, P. & Pybus, O. G. SERAPHIM: studying environmental rasters and phylogenetically informed movements. *Bioinformatics* **32**, 3204-3206 (2016). https://doi.org:10.1093/bioinformatics/btw384

17 Dellicour, S., Vrancken, B., Trovao, N. S., Fargette, D. & Lemey, P. On the importance of negative controls in viral landscape phylogeography. *Virus Evol* **4**, vey023 (2018). https://doi.org:10.1093/ve/vey023

18 McRae, B. H. Isolation by resistance. *Evolution* **60**, 1551-1561 (2006).

19 Baker, R. E. *et al.* Infectious disease in an era of global change. *Nat Rev Microbiol* **20**, 193-205 (2022). https://doi.org:10.1038/s41579-021-00639-z
