# Supplementary material for: West Nile virus spread in Europe: Phylogeographic pattern analysis and key drivers
Source: PLoS Pathog. 2024 Jan 25;20(1):e1011880. doi: 10.1371/journal.ppat.1011880 (PMC10810478; doi:10.1371/journal.ppat.1011880)
Supplement: S1 Table — (DOCX) [file ppat.1011880.s003.docx]

# S1 Table: Sequences and metadata of WNV lineage 2a used in this study

| Accession | country | host | host_ori | date | latitude | longitude | cluster | Gene |
| --- | --- | --- | --- | --- | --- | --- | --- | --- |
| OL840879 | Greece | human | human | 2019.64658 | 41.122439 | 24.3296733 | Cluster B | WGS |
| MN966983 | Romania | human | Homo sapiens | 2015.68575 | 46.1005122 | 27.1810469 | Cluster B | NS5 |
| OL840877 | Greece | human | human | 2020.57377 | 41.7063889 | 26.3013889 | Cluster B | WGS |
| LT996427 | Romania | mosquito | Culex pipiens | 2015.63288 | 44.4361414 | 26.1027202 | Cluster B | NS3 |
| LT996426 | Romania | mosquito | Culex pipiens | 2015.6548 | 44.4361414 | 26.1027202 | Cluster B | NS3 |
| LT996419 | Romania | mosquito | Culex pipiens | 2016.46995 | 44.4361414 | 26.1027202 | Cluster B | NS3 |
| LT996429 | Romania | human | Homo sapiens | 2016.5833 | 44.4361414 | 26.1027202 | Cluster B | NS3 |
| LT996422 | Romania | mosquito | Culex pipiens | 2016.59563 | 44.4361414 | 26.1027202 | Cluster B | NS3 |
| LT996421 | Romania | mosquito | Culex pipiens | 2016.6612 | 44.4361414 | 26.1027202 | Cluster B | NS3 |
| MN481591 | Greece | dog | dog | 2018.6411 | 41.083333 | 25.416667 | Cluster B | WGS |
| OL840876 | Greece | human | human | 2020.60656 | 41.1294444 | 25.0413889 | Cluster B | WGS |
| OL840875 | Greece | human | human | 2019.59178 | 41.1166667 | 24.9125 | Cluster B | WGS |
| KJ883344 | Greece | human | Homo sapiens | 2013.5 | 41.0627778 | 24.8969444 | Cluster B | WGS |
| KJ883345 | Greece | human | Homo sapiens | 2013.5 | 41.130036 | 24.88649 | Cluster B | WGS |
| KJ883341 | Greece | human | Homo sapiens | 2013.5 | 41.130036 | 24.88649 | Cluster B | WGS |
| KJ883343 | Greece | human | Homo sapiens | 2013.5 | 40.9813889 | 24.7994444 | Cluster B | WGS |
| KJ883349 | Greece | human | Homo sapiens | 2013.5 | 40.9822222 | 24.7047222 | Cluster B | WGS |
| KJ883348 | Greece | human | Homo sapiens | 2013.5 | 40.9822222 | 24.7047222 | Cluster B | WGS |
| KJ883342 | Greece | human | Homo sapiens | 2013.5 | 40.9822222 | 24.7047222 | Cluster B | WGS |
| KF179639 | Greece | human | Homo sapiens | 2012.66667 | 40.9080556 | 24.6580556 | Cluster B | WGS |
| MT341472 | Bulgaria | mosquito | Culex pipiens | 2018.58082 | 43.409022 | 24.6180123 | Cluster B | WGS |
| JX860675 | Greece | mosquito | Culex pipiens | 2012.59563 | 41.3504871 | 24.195113 | Cluster B | NS3 |
| LS398545 | Romania | human | Homo sapiens | 2015.5833 | 45.7973912 | 24.1519202 | Cluster B | NS3 |
| MK084830 | Greece | human | Homo sapiens | 2018.66575 | 40.0809716 | 23.978897 | Cluster B | NS3 |
| MK084846 | Greece | mosquito | Culex pipiens | 2018.51781 | 37.9946543 | 23.7994025 | Cluster B | NS3 |
| MK084849 | Greece | mosquito | Culex pipiens | 2018.58082 | 37.9946543 | 23.7994025 | Cluster B | NS3 |
| MK084840 | Greece | human | Homo sapiens | 2018.58082 | 37.9946543 | 23.7994025 | Cluster B | NS3 |
| MK084847 | Greece | mosquito | Culex pipiens | 2018.60548 | 37.9946543 | 23.7994025 | Cluster B | NS3 |
| MK084848 | Greece | mosquito | Culex pipiens | 2018.66575 | 37.9946543 | 23.7994025 | Cluster B | NS3 |
| JN398476 | Greece | bird | Gallus gallus | 2011.50959 | 37.9839412 | 23.7283052 | Cluster B | NS3 |
| MN238866 | Greece | mosquito | Culex pipiens | 2019.52055 | 37.9839412 | 23.7283052 | Cluster B | NS3 |
| MZ333221 | Greece | mosquito | Culex pipiens | 2020.60656 | 41.08683 | 23.54717 | Cluster B | NS3 |
| OL840890 | Greece | mosquito | Culex pipiens | 2020.60656 | 41.0963889 | 23.5022222 | Cluster B | WGS |
| OL840891 | Greece | mosquito | Culex pipiens | 2020.60929 | 41.176068 | 23.391773 | Cluster B | WGS |
| OL840896 | Greece | mosquito | unknown mosquito | 2021.6411 | 41.176068 | 23.391773 | Cluster B | WGS |
| OL840892 | Greece | mosquito | Culex pipiens | 2020.63115 | 41.1955556 | 23.3711111 | Cluster B | WGS |
| OL840883 | Greece | mosquito | unknown mosquito | 2019.53973 | 41.009902 | 23.370939 | Cluster B | WGS |
| OL840893 | Greece | mosquito | Culex pipiens | 2020.55738 | 41.0247222 | 23.3633333 | Cluster B | WGS |
| KU206781 | Bulgaria | human | Homo sapiens | 2015.65206 | 42.698334 | 23.319941 | Cluster B | WGS |
| KY594040 | Greece | human | Homo sapiens | 2010.66575 | 40.6516667 | 23.3041667 | Cluster B | WGS |
| KF537659 | Greece | human | Homo sapiens | 2013.54521 | 40.6516667 | 23.3041667 | Cluster B | NS3 |
| OL840887 | Greece | mosquito | Culex pipiens | 2020.57377 | 41.142305 | 23.213111 | Cluster B | WGS |
| OL840881 | Greece | human | human | 2020.61475 | 41.2077778 | 23.0963889 | Cluster B | WGS |
| OL840886 | Greece | mosquito | Culex pipiens | 2020.60656 | 41.205796 | 23.074921 | Cluster B | WGS |
| MN481589 | Greece | bird | chicken | 2018.53973 | 40.5233333 | 23.0477778 | Cluster B | WGS |
| MN480794 | Greece | mosquito | Culex pipiens | 2018.66575 | 40.7144444 | 23.0416667 | Cluster B | WGS |
| OL840872 | Greece | human | human | 2018.65206 | 40.5466667 | 23.0194444 | Cluster B | WGS |
| MN652880 | Greece | mosquito | Culex pipiens | 2018.58082 | 40.614065 | 22.978366 | Cluster B | WGS |
| KJ883350 | Greece | human | Homo sapiens | 2013.5 | 40.6105556 | 22.9755556 | Cluster B | WGS |
| JN809471 | Greece | bird | Pica pica | 2010.66575 | 40.4737694 | 22.9746167 | Cluster B | NS3 |
| JF719073 | Greece | bird | Pica pica | 2010.66575 | 40.6403167 | 22.9352716 | Cluster B | NS5 |
| MN481593 | Greece | mosquito | unknown mosquito | 2012.63934 | 40.6403167 | 22.9352716 | Cluster B | WGS |
| JX843471 | Greece | bird | Gallus gallus | 2012.65301 | 40.6403167 | 22.9352716 | Cluster B | NS3 |
| MK084829 | Greece | human | Homo sapiens | 2018.58082 | 40.6403167 | 22.9352716 | Cluster B | NS3 |
| MK084828 | Greece | human | Homo sapiens | 2018.66575 | 40.6403167 | 22.9352716 | Cluster B | NS3 |
| MN480792 | Greece | human | Homo sapiens | 2018.58082 | 40.6711111 | 22.9275 | Cluster B | WGS |
| JF917092 | Greece | human | Homo sapiens | 2010.5 | 40.842266 | 22.9204109 | Cluster B | NS3 |
| MK084845 | Greece | mosquito | Culex pipiens | 2018.52877 | 40.6186482 | 22.9179536 | Cluster B | NS3 |
| KJ577739 | Greece | human | Homo sapiens | 2013.5 | 40.6769444 | 22.91 | Cluster B | WGS |
| HQ537483 | Greece | mosquito | Culex pipiens | 2010.5 | 40.8377778 | 22.8986111 | Cluster B | WGS |
| KJ577738 | Greece | human | Homo sapiens | 2013.5 | 40.6569444 | 22.8894444 | Cluster B | WGS |
| OL840889 | Greece | mosquito | unknown mosquito | 2020.60656 | 41.166604 | 22.795426 | Cluster B | WGS |
| MN481595 | Greece | bird | pigeon | 2010.74521 | 40.621643 | 22.752899 | Cluster B | WGS |
| MN481592 | Greece | mosquito | unknown mosquito | 2012.5847 | 40.621643 | 22.752899 | Cluster B | WGS |
| KJ883346 | Greece | human | Homo sapiens | 2013.5 | 40.621643 | 22.752899 | Cluster B | WGS |
| MN652878 | Greece | mosquito | Culex pipiens | 2018.49589 | 40.621643 | 22.752899 | Cluster B | WGS |
| MN481590 | Greece | horse | horse | 2018.53973 | 40.621643 | 22.752899 | Cluster B | WGS |
| OL840884 | Greece | mosquito | unknown mosquito | 2019.57808 | 40.921165 | 22.740333 | Cluster B | WGS |
| MK473443 | Greece | bird | Eurasian magpie | 2017.49589 | 37.6333306 | 22.7333306 | Cluster B | WGS |
| MH549209 | Greece | bird | Pica pica | 2017.49589 | 37.6333306 | 22.7333306 | Cluster B | WGS |
| OL840898 | Greece | mosquito | unknown mosquito | 2021.64384 | 40.708914 | 22.730024 | Cluster B | WGS |
| OL840894 | Greece | mosquito | unknown mosquito | 2021.60274 | 40.619191 | 22.667215 | Cluster B | WGS |
| OL840885 | Greece | mosquito | unknown mosquito | 2019.59726 | 40.660102 | 22.632775 | Cluster B | WGS |
| OL840897 | Greece | mosquito | unknown mosquito | 2021.64384 | 40.559784 | 22.592216 | Cluster B | WGS |
| OL840899 | Greece | mosquito | unknown mosquito | 2021.64384 | 40.783145 | 22.590405 | Cluster B | WGS |
| MW803141 | Serbia | mosquito | Culex pipiens | 2020.5 | 42.8163051 | 22.5649632 | Cluster B | NS5 |
| MK084839 | Greece | human | Homo sapiens | 2018.58082 | 40.763244 | 22.51917 | Cluster B | NS3 |
| OL840888 | Greece | mosquito | unknown mosquito | 2020.57377 | 39.8390171 | 22.5064489 | Cluster B | WGS |
| OL840874 | Greece | human | human | 2019.49863 | 40.280559 | 22.50584 | Cluster B | WGS |
| MK084834 | Greece | human | Homo sapiens | 2018.49589 | 39.6383092 | 22.4160706 | Cluster B | NS3 |
| MN481596 | Greece | bird | chicken | 2011.50959 | 40.7947222 | 22.4116667 | Cluster B | WGS |
| MN481597 | Greece | bird | chicken | 2012.65301 | 40.7947222 | 22.4116667 | Cluster B | WGS |
| MN481594 | Greece | mosquito | unknown mosquito | 2012.6776 | 40.7947222 | 22.4116667 | Cluster B | WGS |
| OL840873 | Greece | human | human | 2018.72877 | 39.7166667 | 22.3958333 | Cluster B | WGS |
| OL840878 | Greece | human | human | 2019.64658 | 40.8252778 | 22.3872222 | Cluster B | WGS |
| MT341470 | Greece | mosquito | Culex pipiens | 2019.49589 | 39.75828 | 22.37297 | Cluster B | WGS |
| OL840882 | Greece | human | human | 2020.66667 | 40.7819444 | 22.275 | Cluster B | WGS |
| OL840895 | Greece | mosquito | unknown mosquito | 2021.60822 | 40.7066667 | 22.265 | Cluster B | WGS |
| MN480795 | Greece | human | Homo sapiens | 2018.58082 | 40.8227778 | 22.2491667 | Cluster B | WGS |
| MN652879 | Greece | mosquito | Culex pipiens | 2018.49589 | 40.818249 | 22.183789 | Cluster B | WGS |
| MN480793 | Greece | human | Homo sapiens | 2018.58082 | 40.818249 | 22.183789 | Cluster B | WGS |
| MK084835 | Greece | human | Homo sapiens | 2018.66575 | 40.517038 | 22.1807197 | Cluster B | NS3 |
| MK084841 | Greece | human | Homo sapiens | 2018.66575 | 40.517038 | 22.1807197 | Cluster B | NS3 |
| MT341471 | Greece | mosquito | Culex pipiens | 2019.49589 | 39.397834 | 22.070087 | Cluster B | WGS |
| OL840871 | Greece | human | human | 2018.6411 | 40.80168 | 22.04398 | Cluster B | WGS |
| OL840880 | Greece | human | human | 2020.51639 | 39.2688889 | 21.8111111 | Cluster B | WGS |
| MH244513 | Slovakia | bird | Eurasian sparrow hawk | 2013.5 | 48.7015059 | 21.6555995 | Cluster A | WGS |
| KJ883347 | Greece | human | Homo sapiens | 2013.5 | 40.7047222 | 21.5219444 | Cluster B | WGS |
| MW561633 | Slovakia | bird | Strix nebulosa lapponica | 2018.87397 | 48.7748988 | 21.2067674 | Cluster B | WGS |
| KT757322 | Serbia | mosquito | Culex pipiens | 2013.5 | 44.8178131 | 20.4568974 | Cluster B | WGS |
| KT757321 | Serbia | mosquito | Culex pipiens | 2013.5 | 44.8178131 | 20.4568974 | Cluster B | WGS |
| KT757320 | Serbia | mosquito | Culex pipiens | 2013.5 | 44.8178131 | 20.4568974 | Cluster B | WGS |
| KT757318 | Serbia | mosquito | Culex pipiens | 2013.5 | 44.8178131 | 20.4568974 | Cluster B | WGS |
| KJ652316 | Serbia | mosquito | Culex pipiens | 2013.5 | 44.8178131 | 20.4568974 | Cluster B | NS3 |
| KJ652314 | Serbia | mosquito | Culex pipiens | 2013.5 | 44.8178131 | 20.4568974 | Cluster B | NS3 |
| MW803139 | Serbia | mosquito | Culex pipiens | 2018.5 | 44.9998526 | 20.4055204 | Cluster B | NS5 |
| DQ116961 | Hungary | bird | goshawk | 2004.5 | 46.35069 | 20.139858 | root | WGS |
| KT757323 | Serbia | mosquito | Culex pipiens | 2013.5 | 45.4094426 | 19.9762492 | Cluster B | WGS |
| KC496016 | Serbia | mosquito | Culex pipiens | 2010.5 | 45.2551338 | 19.8451756 | Cluster A | WGS |
| KC407673 | Serbia | bird | northern goshawk | 2012.5 | 45.2551338 | 19.8451756 | Cluster A | WGS |
| KX375812 | Serbia | human | Homo sapiens | 2013.5 | 45.2551338 | 19.8451756 | Cluster B | WGS |
| MN296400 | Hungary | human | Homo sapiens | 2019.5726 | 47.1716447 | 19.7977516 | Cluster B | NS3 |
| MH021189 | Hungary | human | Homo sapiens | 2017.74795 | 47.4811921 | 19.0602641 | Cluster B | WGS |
| KC496015 | Hungary | horse | horse | 2010.5 | 47.4979937 | 19.0403594 | Cluster B | WGS |
| KT359349 | Hungary | human | Homo sapiens | 2014.5 | 47.4979937 | 19.0403594 | Cluster A | WGS |
| KU058850 | Hungary | human | Homo sapiens | 2014.5 | 47.4979937 | 19.0403594 | Cluster A | NS3 |
| KU058849 | Hungary | human | Homo sapiens | 2014.5 | 47.4979937 | 19.0403594 | Cluster A | NS3 |
| KU058848 | Hungary | human | Homo sapiens | 2014.5 | 47.4979937 | 19.0403594 | Cluster A | NS3 |
| MK224615 | Hungary | human | Homo sapiens | 2018.5 | 47.4979937 | 19.0403594 | Cluster B | NS3 |
| MK224614 | Hungary | human | Homo sapiens | 2018.5 | 47.4979937 | 19.0403594 | Cluster B | NS3 |
| MK224612 | Hungary | human | Homo sapiens | 2018.5 | 47.4979937 | 19.0403594 | Cluster B | NS3 |
| MK224644 | Hungary | human | Homo sapiens | 2018.5 | 47.4979937 | 19.0403594 | Cluster B | NS3 |
| MK224643 | Hungary | human | Homo sapiens | 2018.5 | 47.4979937 | 19.0403594 | Cluster B | NS3 |
| MK224631 | Hungary | human | Homo sapiens | 2018.5 | 47.4979937 | 19.0403594 | Cluster B | NS3 |
| MK224623 | Hungary | human | Homo sapiens | 2018.5 | 47.4979937 | 19.0403594 | Cluster A | NS3 |
| MK224618 | Hungary | human | Homo sapiens | 2018.5 | 47.4979937 | 19.0403594 | Cluster B | NS3 |
| MK224611 | Hungary | human | Homo sapiens | 2018.5 | 47.4979937 | 19.0403594 | Cluster A | NS3 |
| MK224639 | Hungary | human | Homo sapiens | 2018.5 | 47.4979937 | 19.0403594 | Cluster A | NS3 |
| MK224621 | Hungary | human | Homo sapiens | 2018.5 | 47.4979937 | 19.0403594 | Cluster A | NS3 |
| MK224638 | Hungary | human | Homo sapiens | 2018.5 | 47.4979937 | 19.0403594 | Cluster A | NS3 |
| MK224626 | Hungary | human | Homo sapiens | 2018.5 | 47.4979937 | 19.0403594 | Cluster A | NS3 |
| MK224613 | Hungary | human | Homo sapiens | 2018.5 | 47.4979937 | 19.0403594 | Cluster A | NS3 |
| MN255471 | Hungary | human | Homo sapiens | 2019.53425 | 47.4979937 | 19.0403594 | Cluster B | NS3 |
| MN396755 | Hungary | human | Homo sapiens | 2019.58082 | 47.4979937 | 19.0403594 | Cluster A | NS3 |
| MH244512 | Slovakia | bird | northern goshawk | 2013.5 | 48.2131904 | 17.3603744 | Cluster A | WGS |
| MH244511 | Slovakia | bird | northern goshawk | 2013.5 | 48.2131904 | 17.3603744 | Cluster A | WGS |
| MH244510 | Slovakia | bird | northern goshawk | 2014.5 | 48.2131904 | 17.3603744 | Cluster A | WGS |
| MF678435 | Austria | mosquito | Culex pipiens | 2015.66575 | 48.2778951 | 16.9097882 | Cluster A | NS5 |
| KM203863 | Czech_Republic | mosquito | Culex modestus | 2013.6 | 48.8001413 | 16.8039515 | Cluster A | WGS |
| KM203862 | Czech_Republic | mosquito | Culex modestus | 2013.6 | 48.8001413 | 16.8039515 | Cluster A | WGS |
| MF678440 | Austria | mosquito | Culex pipiens | 2015.49589 | 48.3189487 | 16.6429437 | Cluster A | NS5 |
| KM203860 | Czech_Republic | mosquito | Culex modestus | 2013.6 | 48.8068577 | 16.6383914 | Cluster A | WGS |
| KM203861 | Czech_Republic | mosquito | Culex modestus | 2013.6 | 48.8068577 | 16.6383914 | Cluster A | WGS |
| MF678434 | Austria | mosquito | Culex pipiens | 2015.58082 | 48.2269664 | 16.4944845 | Cluster A | NS5 |
| MF678444 | Austria | mosquito | Culex pipiens | 2015.58082 | 48.2006384 | 16.4269481 | Cluster A | NS5 |
| MF678445 | Austria | mosquito | Culex pipiens | 2015.58082 | 48.2006384 | 16.4269481 | Cluster A | NS5 |
| MF678432 | Austria | mosquito | Culex pipiens | 2015.58082 | 48.2798151 | 16.4121346 | Cluster A | NS5 |
| KF179640 | Austria | bird | goshawk | 2008.5 | 48.2083537 | 16.3725042 | Cluster A | WGS |
| KP780838 | Austria | bird | Nestor notabilis | 2009.70685 | 48.2083537 | 16.3725042 | Cluster A | WGS |
| KP780839 | Austria | bird | Nestor notabilis | 2011.19726 | 48.2083537 | 16.3725042 | Cluster A | WGS |
| KP109692 | Austria | mosquito | Culex pipiens | 2014.5 | 48.2083537 | 16.3725042 | Cluster A | WGS |
| KP109691 | Austria | human | Homo sapiens | 2014.5 | 48.2083537 | 16.3725042 | Cluster A | WGS |
| KM659876 | Austria | human | Homo sapiens | 2014.5 | 48.2083537 | 16.3725042 | Cluster A | WGS |
| KP780840 | Austria | bird | Nestor notabilis | 2014.63562 | 48.2083537 | 16.3725042 | Cluster A | NS3 |
| MF984345 | Austria | bird | falcon | 2015.5 | 48.2083537 | 16.3725042 | Cluster A | WGS |
| MF984342 | Austria | human | Homo sapiens | 2015.5 | 48.2083537 | 16.3725042 | Cluster A | WGS |
| MF984340 | Austria | human | Homo sapiens | 2015.5 | 48.2083537 | 16.3725042 | Cluster A | WGS |
| MF984337 | Austria | human | Homo sapiens | 2015.5 | 48.2083537 | 16.3725042 | Cluster A | WGS |
| MF984344 | Austria | bird | goshawk | 2015.5 | 48.2083537 | 16.3725042 | Cluster A | WGS |
| MF984341 | Austria | human | Homo sapiens | 2015.5 | 48.2083537 | 16.3725042 | Cluster A | WGS |
| MF984343 | Austria | human | Homo sapiens | 2015.5 | 48.2083537 | 16.3725042 | Cluster A | WGS |
| MF984338 | Austria | human | Homo sapiens | 2015.5 | 48.2083537 | 16.3725042 | Cluster A | WGS |
| MF984339 | Austria | human | Homo sapiens | 2015.5 | 48.2083537 | 16.3725042 | Cluster A | WGS |
| MF984346 | Austria | human | Homo sapiens | 2016.5 | 48.2083537 | 16.3725042 | Cluster A | WGS |
| MF984347 | Austria | human | Homo sapiens | 2016.5 | 48.2083537 | 16.3725042 | Cluster A | WGS |
| MF984350 | Austria | horse | horse | 2016.5 | 48.2083537 | 16.3725042 | Cluster A | WGS |
| MF984348 | Austria | human | Homo sapiens | 2016.5 | 48.2083537 | 16.3725042 | Cluster A | WGS |
| MF984351 | Austria | mosquito | Culex pipiens | 2016.5 | 48.2083537 | 16.3725042 | Cluster A | WGS |
| MF984349 | Austria | horse | horse | 2016.5 | 48.2083537 | 16.3725042 | Cluster A | WGS |
| MF984352 | Austria | mosquito | Culex pipiens | 2016.5 | 48.2083537 | 16.3725042 | Cluster A | WGS |
| MF678441 | Austria | mosquito | Culex pipiens | 2015.58082 | 48.1777617 | 16.3307487 | Cluster A | NS5 |
| EF116943 | Hungary | bird | Accipiter nisus | 2005.5 | 48.1820863 | 16.3005958 | Cluster A | NS5 |
| MF678439 | Austria | mosquito | Culex pipiens | 2015.49589 | 48.2018182 | 16.2589955 | Cluster A | NS5 |
| MF678442 | Austria | mosquito | Culex pipiens | 2015.58082 | 48.2018182 | 16.2589955 | Cluster A | NS5 |
| MF678443 | Austria | mosquito | Culex pipiens | 2015.58082 | 48.178541 | 16.2529858 | Cluster A | NS5 |
| GU566737 | Austria | bird | Accipiter gentilis | 2008.5 | 48.1873864 | 15.8440588 | Cluster A | NS5 |
| GU566738 | Austria | bird | Accipiter gentilis | 2008.5 | 48.1873864 | 15.8440588 | Cluster A | NS5 |
| GU566739 | Austria | bird | Accipiter gentilis | 2008.5 | 48.1873864 | 15.8440588 | Cluster A | NS5 |
| HM015884 | Austria | bird | Accipiter gentilis | 2009.5 | 48.1873864 | 15.8440588 | Cluster A | NS5 |
| GU566740 | Austria | bird | Accipiter gentilis | 2009.5 | 48.1873864 | 15.8440588 | Cluster A | NS5 |
| GU580891 | Austria | bird | Falcon | 2008.5 | 47.2174206 | 15.6221694 | Cluster A | NS5 |
| KP780837 | Austria | bird | Nestor notabilis | 2008.65847 | 46.6785742 | 14.9069497 | Cluster A | WGS |
| MK947396 | Slovenia | mosquito | unknown mosquito | 2018.5 | 46.0499803 | 14.5068602 | Cluster A | WGS |
| MK947397 | Slovenia | human | Homo sapiens | 2018.6411 | 46.0499803 | 14.5068602 | Cluster B | WGS |
| LR743448 | Germany | bird | Humboldt-Penguin | 2019.68767 | 51.7567447 | 14.3357307 | Cluster A | WGS |
| LR743431 | Germany | bird | Eurasian Golden Plover | 2019.67397 | 51.270139 | 14.0948628 | Cluster A | WGS |
| LR743421 | Germany | bird | Great Tit | 2019.66027 | 51.0493286 | 13.7381437 | Cluster A | WGS |
| LR743430 | Germany | bird | Blue Tit | 2019.6 | 51.0999765 | 13.6767995 | Cluster A | WGS |
| LR989888 | Germany | bird | Larus crassirostris | 2019.58082 | 52.5023038 | 13.5291672 | Cluster A | WGS |
| LR989885 | Germany | bird | Larus crassirostris | 2019.58082 | 52.5023038 | 13.5291672 | Cluster A | WGS |
| LR743443 | Germany | bird | Snowy Owl | 2018.67671 | 52.5029358 | 13.5205456 | Cluster A | WGS |
| LR743424 | Germany | bird | Snowy Owl | 2019.58904 | 52.5029358 | 13.5205456 | Cluster A | WGS |
| LR743428 | Germany | bird | Snowy Owl | 2019.6274 | 52.5029358 | 13.5205456 | Cluster A | WGS |
| LR743423 | Germany | bird | Goshawk | 2019.66301 | 52.5029358 | 13.5205456 | Cluster A | WGS |
| LR743455 | Germany | mosquito | unknown mosquito | 2019.70959 | 52.5029358 | 13.5205456 | Cluster A | WGS |
| LR743447 | Germany | mosquito | unknown mosquito | 2019.72877 | 52.5029358 | 13.5205456 | Cluster A | WGS |
| MH910045 | Germany | human | Homo sapiens | 2018.58082 | 52.5170365 | 13.3888599 | Cluster A | WGS |
| MH986055 | Germany | bird | Turdus merula | 2018.58082 | 52.5170365 | 13.3888599 | Cluster A | WGS |
| MH986056 | Germany | bird | Turdus merula | 2018.7219 | 52.5170365 | 13.3888599 | Cluster A | WGS |
| LR743427 | Germany | bird | Goshawk | 2019.53151 | 52.5170365 | 13.3888599 | Cluster A | WGS |
| LR743426 | Germany | bird | Snowy Owl | 2019.66301 | 52.5170365 | 13.3888599 | Cluster A | WGS |
| KT364499 | Italy | mosquito | Culex pipiens | 2011.5 | 46.0433627 | 13.2362187 | Cluster A | NS3 |
| MH974750 | Germany | bird | Accipiter gentilis | 2018.6918 | 52.5138394 | 13.2135845 | Cluster A | NS5 |
| LR743435 | Germany | bird | Goshawk | 2019.63288 | 52.5873198 | 12.9920587 | Cluster A | WGS |
| LR743445 | Germany | bird | Great Grey Owl | 2019.67123 | 50.8322608 | 12.9252977 | Cluster A | WGS |
| LR743452 | Germany | bird | Great Grey Owl | 2019.67397 | 50.8322608 | 12.9252977 | Cluster A | WGS |
| LR743422 | Germany | bird | Goshawk | 2019.64932 | 52.9243859 | 12.8092919 | Cluster A | WGS |
| LR743436 | Germany | bird | Goshawk | 2018.69041 | 51.5911653 | 12.5856428 | Cluster A | WGS |
| MH974752 | Germany | bird | Accipiter gentilis | 2018.7164 | 51.5911653 | 12.5856428 | Cluster A | NS5 |
| LR743454 | Germany | bird | Great Tit | 2019.68767 | 51.5911653 | 12.5856428 | Cluster A | WGS |
| MN794938 | Germany | bird | House sparrow | 2019.6548 | 51.4224434 | 12.5645728 | Cluster A | WGS |
| LR743425 | Germany | bird | Snowy Owl | 2019.50137 | 51.9064957 | 12.5417127 | Cluster A | WGS |
| LR743444 | Germany | bird | Great Grey Owl | 2019.59452 | 51.9064957 | 12.5417127 | Cluster A | WGS |
| JN858070 | Italy | human | Homo sapiens | 2011.66575 | 41.8933203 | 12.4829321 | Cluster A | WGS |
| LR743453 | Germany | horse | Horse | 2019.61644 | 51.4617493 | 12.452697 | Cluster A | WGS |
| LR743450 | Germany | bird | Chilean Flamingo | 2019.68767 | 51.3406321 | 12.3747329 | Cluster A | WGS |
| MN794935 | Germany | human | Human | 2019.69041 | 51.3406321 | 12.3747329 | Cluster A | WGS |
| MW142227 | Germany | human | Homo sapiens | 2020.58197 | 51.3406321 | 12.3747329 | Cluster A | WGS |
| MW142226 | Germany | human | Homo sapiens | 2020.58197 | 51.3406321 | 12.3747329 | Cluster A | WGS |
| MW142225 | Germany | human | Homo sapiens | 2020.58197 | 51.3406321 | 12.3747329 | Cluster A | WGS |
| MW142223 | Germany | human | Homo sapiens | 2020.58197 | 51.3406321 | 12.3747329 | Cluster A | WGS |
| MW142224 | Germany | human | Homo sapiens | 2020.58197 | 51.3406321 | 12.3747329 | Cluster A | WGS |
| LR743456 | Germany | bird | House Sparrow | 2019.64932 | 51.4390092 | 12.3714685 | Cluster A | WGS |
| MN939561 | Italy | human | Homo sapiens | 2018.6137 | 45.4371908 | 12.3345898 | Cluster A | WGS |
| JX878386 | Italy | mosquito | Culex pipiens | 2012.5 | 44.9772062 | 12.2741904 | Cluster B | NS3 |
| KF647250 | Italy | human | Homo sapiens | 2013.61096 | 44.9772062 | 12.2741904 | Cluster A | WGS |
| KF647249 | Italy | human | Homo sapiens | 2013.61096 | 44.9772062 | 12.2741904 | Cluster A | WGS |
| KF588365 | Italy | human | Homo sapiens | 2013.61096 | 44.9772062 | 12.2741904 | Cluster A | WGS |
| KF647248 | Italy | human | Homo sapiens | 2013.63014 | 44.9772062 | 12.2741904 | Cluster A | WGS |
| KF647252 | Italy | human | Homo sapiens | 2013.64932 | 44.9772062 | 12.2741904 | Cluster A | WGS |
| KF823805 | Italy | human | Homo sapiens | 2013.64932 | 44.9772062 | 12.2741904 | Cluster A | WGS |
| MN939557 | Italy | human | Homo sapiens | 2016.54372 | 44.9772062 | 12.2741904 | Cluster A | WGS |
| MN939564 | Italy | human | Homo sapiens | 2016.60109 | 44.9772062 | 12.2741904 | Cluster A | WGS |
| MN939558 | Italy | human | Homo sapiens | 2016.60109 | 44.9772062 | 12.2741904 | Cluster A | WGS |
| MN939559 | Italy | human | Homo sapiens | 2016.63934 | 44.9772062 | 12.2741904 | Cluster A | WGS |
| OP561457 | Italy | human | Homo sapiens | 2019.631 | 44.9772062 | 12.2741904 | Cluster A | WGS |
| KP407865 | Italy | bird | collared dove | 2011.5 | 45.657618 | 12.266908 | Cluster A | NS3 |
| OP561456 | Italy | human | Homo sapiens | 2019.611 | 45.657618 | 12.266908 | Cluster A | WGS |
| LR743449 | Germany | bird | Goshawk | 2019.67397 | 51.6181969 | 12.2635906 | Cluster A | WGS |
| MN939562 | Italy | human | Homo sapiens | 2018.65206 | 45.8066914 | 12.2063158 | Cluster A | WGS |
| KT364505 | Italy | mosquito | Culex pipiens | 2013.5 | 45.842483 | 12.100108 | Cluster A | NS3 |
| KT364501 | Italy | mosquito | Culex pipiens | 2013.5 | 45.842483 | 12.100108 | Cluster B | NS3 |
| KT364503 | Italy | mosquito | Culex pipiens | 2013.5 | 45.842483 | 12.100108 | Cluster A | NS3 |
| KT364502 | Italy | mosquito | Culex pipiens | 2013.5 | 45.842483 | 12.100108 | Cluster A | NS3 |
| KT364504 | Italy | mosquito | Culex pipiens | 2013.5 | 45.842483 | 12.100108 | Cluster A | NS3 |
| LR743433 | Germany | bird | Goshawk | 2018.66575 | 51.6796187 | 12.0611585 | Cluster A | WGS |
| LR743457 | Germany | bird | Goshawk | 2019.67671 | 51.3564413 | 11.996148 | Cluster A | WGS |
| LR743442 | Germany | bird | Blue Tit | 2019.6 | 51.4825041 | 11.9705452 | Cluster A | WGS |
| LR743446 | Germany | bird | Coconut Lorikeet | 2019.63014 | 51.4825041 | 11.9705452 | Cluster A | WGS |
| MH924836 | Germany | bird | Strix nebulosa | 2018.63836 | 51.4728617 | 11.9023342 | Cluster A | NS3 |
| LR743429 | Germany | bird | Tawny Owl | 2018.71507 | 51.406202 | 11.8889417 | Cluster A | WGS |
| KF647251 | Italy | human | Homo sapiens | 2013.63014 | 45.4077172 | 11.8734455 | Cluster A | WGS |
| OP561453 | Italy | human | Homo sapiens | 2019.534 | 45.4064823 | 11.8212057 | Cluster A | WGS |
| OP561458 | Italy | human | Homo sapiens | 2019.534 | 45.4064823 | 11.8212057 | Cluster A | WGS |
| MH974754 | Germany | bird | Strix nebulosa | 2018.6973 | 48.172047 | 11.8090838 | Cluster A | NS5 |
| LR743437 | Germany | bird | Great Grey Owl | 2018.66301 | 48.1667467 | 11.803713 | Cluster A | WGS |
| LR743434 | Germany | bird | Great Grey Owl | 2018.67123 | 48.1667467 | 11.803713 | Cluster A | NS3 |
| KP407864 | Italy | mosquito | unknown mosquito | 2012.5 | 45.0696016 | 11.7715269 | Cluster A | NS3 |
| KP407863 | Italy | mosquito | unknown mosquito | 2012.5 | 45.0696016 | 11.7715269 | Cluster A | NS3 |
| KT364506 | Italy | mosquito | Culex pipiens | 2014.5 | 45.0696016 | 11.7715269 | Cluster A | NS3 |
| KT207792 | Italy | mosquito | unknown mosquito | 2014.6274 | 45.0696016 | 11.7715269 | Cluster A | WGS |
| MN794937 | Germany | bird | Blackbird | 2019.66849 | 52.024109 | 11.7702539 | Cluster A | WGS |
| LR743458 | Germany | bird | Snowy Owl | 2019.67123 | 52.1315889 | 11.6399609 | Cluster A | WGS |
| LR743451 | Germany | bird | Snowy Owl | 2019.67397 | 52.1315889 | 11.6399609 | Cluster A | WGS |
| KU573081 | Italy | bird | Pica pica | 2013.5 | 44.8494142 | 11.6172101 | Cluster A | WGS |
| KU573080 | Italy | mosquito | Culex pipiens | 2013.5 | 44.8494142 | 11.6172101 | Cluster A | WGS |
| KU573082 | Italy | bird | Crow | 2013.5 | 44.8494142 | 11.6172101 | Cluster A | WGS |
| KT364508 | Italy | mosquito | Culex pipiens | 2014.5 | 45.554241 | 11.548145 | Cluster A | NS3 |
| MN939560 | Italy | human | Homo sapiens | 2016.6776 | 45.6348591 | 11.4063543 | Cluster A | WGS |
| KP789956 | Italy | human | Homo sapiens | 2014.66301 | 45.4384958 | 10.9924122 | Cluster A | WGS |
| KP789955 | Italy | human | Homo sapiens | 2014.66301 | 45.4384958 | 10.9924122 | Cluster A | WGS |
| MN939563 | Italy | human | Homo sapiens | 2018.70959 | 45.4384958 | 10.9924122 | Cluster A | WGS |
| OP561454 | Italy | human | Homo sapiens | 2020.631 | 45.4384958 | 10.9924122 | Cluster A | WGS |
| OP561455 | Italy | human | Homo sapiens | 2019.592 | 45.4384958 | 10.9924122 | Cluster A | WGS(PARTIAL) |
| OP561454 | Italy | human | Homo sapiens | 2020.631 | 45.4384958 | 10.9924122 | Cluster A | WGS(PARTIAL) |
| OP561453 | Italy | human | Homo sapiens | 2019.592 | 45.4384958 | 10.9924122 | Cluster A | WGS |
| KT364507 | Italy | mosquito | Culex pipiens | 2014.5 | 45.447869 | 10.982577 | Cluster A | NS3 |
| KU573083 | Italy | mosquito | Culex pipiens | 2013.5 | 44.6501718 | 10.8867129 | Cluster A | WGS |
| KP789960 | Italy | human | Homo sapiens | 2013.66849 | 45.1692628 | 10.6708365 | Cluster A | WGS |
| KF823806 | Italy | human | Homo sapiens | 2013.74521 | 45.1692628 | 10.6708365 | Cluster A | WGS |
| KP789957 | Italy | human | Homo sapiens | 2014.5 | 45.2208641 | 10.037038 | Cluster A | WGS |
| KP789954 | Italy | human | Homo sapiens | 2014.5 | 45.2208641 | 10.037038 | Cluster A | WGS |
| MN794939 | Germany | bird | Dunnock | 2019.6548 | 53.550341 | 10.000654 | Cluster A | WGS |
| KP789959 | Italy | human | Homo sapiens | 2014.5 | 45.0368547 | 9.13782508 | Cluster A | WGS |
| KP789958 | Italy | human | Homo sapiens | 2014.5 | 45.0368547 | 9.13782508 | Cluster A | WGS |
| KP789953 | Italy | human | Homo sapiens | 2014.5 | 45.0368547 | 9.13782508 | Cluster A | WGS |
| KP407867 | Italy | bird | goshawk | 2012.5 | 40.0895341 | 8.830462 | Cluster A | NS3 |
| OP762596 | Netherlands | bird | House sparrow | 2020.735 | 52.0955124 | 5.01253414 | Cluster A | WGS |
| OP762597 | Netherlands | bird | Chicken | 2020.795 | 52.1057167 | 5.01215982 | Cluster A | WGS |
| MW036633 | Netherlands | mosquito | Culex pipiens | 2020.661 | 52.1342798 | 5.0064058 | Cluster A | WGS |
| OP762593 | Netherlands | mosquito | Culex pipiens | 2020.705 | 52.135513 | 5.003988 | Cluster A | WGS |
| OP762595 | Netherlands | bird | Common chiffchaff | 2020.738 | 52.1361694 | 5.00349379 | Cluster A | WGS |
| MW036634 | Netherlands | mosquito | Culex pipiens | 2020.642 | 52.1357615 | 5.0034393 | Cluster A | WGS |
| OP762594 | Netherlands | mosquito | Culex pipiens | 2020.705 | 52.13647 | 5.003069 | Cluster A | WGS |
| OP762592 | Netherlands | mosquito | Culex pipiens | 2020.705 | 52.135254 | 5.002699 | Cluster A | WGS |
| MT863560 | France | bird | Buteo buteo | 2018.68493 | 48.8588897 | 2.32004102 | Cluster A | WGS |
| MT863561 | France | bird | Accipiter gentilis | 2018.76164 | 48.8588897 | 2.32004102 | Cluster A | WGS |
| OM037672 | Spain | bird | Accipiter gentilis | 2020.66575 | 41.1134734 | 1.2379323 | Cluster A | WGS |
| OM037673 | Spain | bird | Accipiter gentilis | 2020.66575 | 41.1134734 | 1.2379323 | Cluster A | WGS |
| OM037670 | Spain | bird | Accipiter gentilis | 2017.66575 | 41.746975 | 0.594472 | Cluster A | WGS |
| OM037671 | Spain | bird | Accipiter gentilis | 2020.66575 | 41.6701857 | 0.5381169 | Cluster A | WGS |
