# Supplementary material for: West Nile virus spread in Europe: Phylogeographic pattern analysis and key drivers
Source: PLoS Pathog. 2024 Jan 25;20(1):e1011880. doi: 10.1371/journal.ppat.1011880 (PMC10810478; doi:10.1371/journal.ppat.1011880)
Supplement: S4 Table — (DOCX) [file ppat.1011880.s006.docx]

# S4 Table: Results of drivers on viral dispersal direction*

| Group | Risk Factor | Ns3 | | Ns5 | | ClusterA | | ClusterB | |
| --- | --- | --- | --- | --- | --- | --- | --- | --- | --- |
|  |  | Remain_in | Leave_from | Remain_in | Leave_from | Remain_in | Leave_from | Remain_in | Leave_from |
| Climate and Weather | Annual mean temperature | 0.3 | 0.3 | 0.4 | 0.7 | 0.2 | 0.1 | 1.4 | 1.6 |
| Climate and Weather | Annual total precipitation | 0.1 | 0 | 0.1 | 0.4 | 0 | 0 | 0.6 | 0 |
| Land Use and Cover | Deciduous Broadleaf Trees | 0.2 | 0.1 | 0.2 | 0.2 | 0 | 0.8 | 1.9 | 0.2 |
| Land Use and Cover | Evergreen Broadleaf Trees | 0 | 0.2 | 0 | 0 | 0 | 0 | 0 | 0 |
| Land Use and Cover | Mixed/Other Trees | 0.4 | 0.2 | 0.9 | 0.3 | 0.1 | 0.9 | 0.1 | 0.6 |
| Land Use and Cover | Shrubs | 1.9 | 0.3 | 2.1 | 0.4 | 0.9 | 1 | 1.4 | 0.4 |
| Land Use and Cover | Evergreen/Deciduous Needleleaf Trees | 1 | 0.2 | 3 | 0.1 | 0.9 | 0.6 | 0.2 | 0.8 |
| Land Use and Cover | Cropland | >100 | 1 | 19 | 2.4 | >100 | 0.4 | >100 | 6.1 |
| Land Use and Cover | Urbanization of cropland | >100 | 1.6 | >100 | 0.8 | >100 | 2.4 | >100 | 3.3 |
| Land Use and Cover | Urbanization of pasture | 11.5 | 0.8 | 3.2 | 0.6 | 1.3 | 0.7 | >100 | 2.2 |
| Land Use and Cover | Urbanization of secondary land | 19 | 0.6 | 13.3 | 0.4 | 5.2 | 1.5 | 6.1 | 2.6 |
| Land Use and Cover | Urbanization of primary land | 0 | 0 | 0 | 0 | 0 | 0 | 0 | 0 |
| Land Use and Cover | Pasture | 2.4 | 0 | 1.9 | 0 | 0.1 | 0 | >100 | 0.1 |
| Land Use and Cover | Primary land | 0.7 | 0.6 | 0.9 | 1.4 | 0.3 | 1.5 | 0.8 | 0.2 |
| Land Use and Cover | Secondary land | 0.7 | 0 | 0.9 | 0 | 0.2 | 0.1 | 0.6 | 0.3 |
| Land Use and Cover | Urban land | >100 | >100 | >100 | >100 | >100 | >100 | >100 | >100 |
| Land Use and Cover | Cultivated and Managed Vegetation | 99 | 0.5 | 5.2 | 1.5 | 32.3 | 0.4 | 32.3 | 1.2 |
| Land Use and Cover | Regularly Flooded Vegetation | 0.4 | 0.5 | 0.3 | 0.4 | 0.4 | 0.4 | 0.3 | 0.5 |
| Land Use and Cover | Herbaceous Vegetation | 0.8 | 0.3 | 1.6 | 0.1 | 0.2 | 1.4 | 0.1 | 1.1 |
| Land Use and Cover | Open water | 0 | 0.4 | 0.2 | 0.9 | 0 | 0.8 | 0.1 | 0.4 |
| Land Use and Cover | Lake_river_reservoir | 0.6 | 0.2 | 0.5 | 0.3 | 0.3 | 0.6 | 0.4 | 0.5 |
| Land Use and Cover | Wetland_combine | 99 | 0.2 | >100 | 0.2 | >100 | 0.2 | 18 | 0.4 |
| Land Use and Cover | Wetland_other | >100 | 0.2 | >100 | 0.2 | >100 | 0.2 | 11 | 0.4 |
| Land Use and Cover | Wetland concentration | 2.1 | 1.0 | 9.0 | 3.2 | 6.7 | 1.2 | 0.9 | 1.6 |
| Topography | Elevation | 0 | 0 | 0.1 | 0 | 0 | 0 | 0.2 | 0 |
| Socio-economic | GDP | >100 | >100 | >100 | >100 | >100 | >100 | >100 | >100 |
| Socio-economic | Human population | >100 | >100 | >100 | >100 | >100 | >100 | >100 | >100 |
| Biodiversity | Livestock count | 24 | 1.1 | 32.3 | 0.8 | 6.7 | 0.4 | >100 | 9 |
| Biodiversity | Mammal species richness | 99 | 0.5 | 4 | 0.4 | 5.7 | 0.3 | 11.5 | 1.4 |
| Biodiversity | Flyway_Anseriformes | 14 | 0.3 | 12 | 0.1 | 10.1 | 0.6 | 0 | 0.1 |
| Biodiversity | Flyway_Apodiformes | 1 | 0.5 | 1.8 | 0.4 | 1.9 | 0.4 | 0.3 | 1.1 |
| Biodiversity | Flyway_Passeriformes | 49 | 1.3 | 3 | 2.2 | 13.3 | 6.1 | 6.7 | 0.2 |
| Biodiversity | Birds Directive | >100 | 1.9 | 24 | 1.7 | 15.7 | 2.1 | >100 | 2.8 |
| Biodiversity | Birds and Habitats Directives | 2.4 | 0.3 | 1.9 | 0.3 | 2.3 | 0.4 | 0.4 | 0.8 |
| Biodiversity | Habitats Directive | >100 | 1.6 | 49 | 2.4 | 15.7 | 2.4 | >100 | 2.8 |
| Biodiversity | Richness of forest-related species and habitats | 0.3 | 0.5 | 0.3 | 1 | 1.1 | 1 | 0 | 0.2 |
| Biodiversity | Culex pipiens status | 0 | 0 | 0 | 0 | 0 | 0 | 0 | 0 |

*Bayes factor (BF>20 in red suggest significant) supports for the association between value of potential factors (n=37) and tree node locations (remain in, leave from) as well as dispersal durations and environmental distances computed for each branch (resistance, conductance). Analyses were performed on data of NS3, NS5 and also separated for Cluster A and B.
