# Supplementary material for: West Nile virus spread in Europe: Phylogeographic pattern analysis and key drivers
Source: PLoS Pathog. 2024 Jan 25;20(1):e1011880. doi: 10.1371/journal.ppat.1011880 (PMC10810478; doi:10.1371/journal.ppat.1011880)
Supplement: S5 Table — (DOCX) [file ppat.1011880.s007.docx]

# S5 Table: Results of drivers on viral dispersal velocity*

| Group | Risk Factor | k | Ns3 | | Ns5 | | Cluster A | | Cluster B | |
| --- | --- | --- | --- | --- | --- | --- | --- | --- | --- | --- |
|  |  |  | Conductance | Resistance | Conductance | Resistance | Conductance | Resistance | Conductance | Resistance |
| Climate and Weather | Anunal mean temperature | 10 | 0.6 | 2.3 | 2.8 | 2.3 | 0.8 | 2.1 | 1.2 | 1.1 |
| Climate and Weather | Anunal mean temperature | 100 | 0.5 | 1.9 | 2.2 | 1.9 | 0.9 | 2.7 | 1.2 | 1.0 |
| Climate and Weather | Anunal mean temperature | 1000 | 1.1 | 1.4 | 2.0 | 2.6 | 1.1 | 1.8 | 1.4 | 1.9 |
| Climate and Weather | Annual total precipitation | 10 | 1.3 | 1.3 | 1.4 | 1.3 | 0.3 | 1.8 | 0.7 | 1.5 |
| Climate and Weather | Annual total precipitation | 100 | 1.4 | 1.0 | 2.8 | 1.5 | 0.7 | 2.3 | 1.4 | 2.8 |
| Climate and Weather | Annual total precipitation | 1000 | 1.4 | 1.0 | 1.9 | 1.5 | 0.9 | 3.8 | 1.8 | 3.3 |
| Land Use and Cover | Deciduous Broadleaf Trees | 10 | 0.9 | 3.5 | 1.9 | 19.0 | 0.3 | 8.1 | 0.8 | 1.6 |
| Land Use and Cover | Deciduous Broadleaf Trees | 100 | 0.7 | 3.5 | 6.7 | 5.7 | 0.5 | 13.3 | 0.7 | 1.9 |
| Land Use and Cover | Deciduous Broadleaf Trees | 1000 | 0.3 | 3.2 | 0.7 | 4.9 | 0.4 | 8.1 | 0.7 | 1.5 |
| Land Use and Cover | Evergreen Broadleaf Trees | 10 | 2.4 | 2.7 | 1.8 | 1.8 | 0.6 | 0.4 | 0.7 | 0.9 |
| Land Use and Cover | Evergreen Broadleaf Trees | 100 | 2.2 | 3.3 | 1.5 | 1.6 | 0.6 | 0.7 | 0.9 | 0.8 |
| Land Use and Cover | Evergreen Broadleaf Trees | 1000 | 2.4 | 3.8 | 1.3 | 2.1 | 0.5 | 0.7 | 1.3 | 0.9 |
| Land Use and Cover | Mixed/Other Trees | 10 | 3.3 | 1.4 | 0.4 | 0.9 | 1.3 | 2.6 | 1.1 | 1.3 |
| Land Use and Cover | Mixed/Other Trees | 100 | 3.5 | 1.7 | 0.3 | 1.3 | 0.5 | 2.6 | 2.1 | 1.4 |
| Land Use and Cover | Mixed/Other Trees | 1000 | 0.7 | 1.1 | 0.1 | 2.7 | 0.3 | 2.2 | 0.7 | 1.2 |
| Land Use and Cover | Shrubs | 10 | 1.4 | 0.8 | 9.0 | 1.3 | 0.6 | 0.7 | 1.9 | 0.8 |
| Land Use and Cover | Shrubs | 100 | 0.5 | 0.7 | 2.0 | 0.6 | 0.8 | 0.6 | 1.1 | 1.7 |
| Land Use and Cover | Shrubs | 1000 | 0.8 | 0.5 | 1.8 | 0.9 | 0.9 | 0.8 | 1.5 | 0.7 |
| Land Use and Cover | Evergreen/Deciduous Needleleaf Trees | 10 | 3.2 | 0.4 | 0.4 | 0.6 | 4.3 | 0.9 | 0.9 | 3.2 |
| Land Use and Cover | Evergreen/Deciduous Needleleaf Trees | 100 | 2.8 | 0.5 | 0.2 | 0.6 | 2.4 | 0.5 | 1.1 | 2.6 |
| Land Use and Cover | Evergreen/Deciduous Needleleaf Trees | 1000 | 2.8 | 0.7 | 0.3 | 1.9 | 2.4 | 0.8 | 1.1 | 2.6 |
| Land Use and Cover | Cropland | 10 | 5.3 | 1.7 | 3.5 | 4.3 | 5.7 | 0.8 | 1.4 | 1.9 |
| Land Use and Cover | Cropland | 100 | 4.9 | 1.4 | 32.3 | 1.5 | 5.3 | 1.0 | 1.9 | 1.2 |
| Land Use and Cover | Cropland | 1000 | 1.0 | 1.0 | 3.0 | 1.6 | 1.2 | 1.3 | 1.0 | 1.0 |
| Land Use and Cover | Urbanization of cropland | 10 | 1.9 | 2.3 | 2.1 | 1.6 | 1.0 | 0.8 | 0.9 | 1.0 |
| Land Use and Cover | Urbanization of cropland | 100 | 2.2 | 0.6 | 3.2 | 0.2 | 2.4 | 1.3 | 1.0 | 1.7 |
| Land Use and Cover | Urbanization of cropland | 1000 | 2.4 | 0.4 | 2.6 | 0.2 | 1.9 | 1.3 | 1.8 | 1.6 |
| Land Use and Cover | Urbanization of pasture | 10 | 1.8 | 2.7 | 2.7 | 2.0 | 0.6 | 1.3 | 0.9 | 1.2 |
| Land Use and Cover | Urbanization of pasture | 100 | 6.1 | 1.9 | 5.7 | 1.1 | 0.8 | 1.7 | 0.8 | 1.1 |
| Land Use and Cover | Urbanization of pasture | 1000 | 5.7 | 0.9 | 8.1 | 0.9 | 1.5 | 1.1 | 2.1 | 1.0 |
| Land Use and Cover | Urbanization of secondary land | 10 | 1.9 | 2.0 | 2.1 | 2.2 | 0.6 | 1.4 | 0.9 | 0.9 |
| Land Use and Cover | Urbanization of secondary land | 100 | 1.4 | 1.3 | 2.2 | 1.6 | 1.9 | 0.8 | 0.7 | 1.3 |
| Land Use and Cover | Urbanization of secondary land | 1000 | 0.9 | 1.2 | 0.4 | 0.8 | 1.8 | 0.8 | 0.6 | 0.9 |
| Land Use and Cover | Urbanization of primary land | 10 | 4.3 | 2.1 | 3.0 | 1.8 | 0.6 | 0.9 | 1.2 | 0.9 |
| Land Use and Cover | Urbanization of primary land | 100 | 4.3 | 2.7 | 4.6 | 2.1 | 0.5 | 0.9 | 1.8 | 1.0 |
| Land Use and Cover | Urbanization of primary land | 1000 | 2.8 | 2.4 | 5.7 | 1.4 | 0.5 | 1.9 | 1.3 | 0.9 |
| Land Use and Cover | Pasture | 10 | 8.1 | 0.9 | 2.0 | 3.0 | 2.3 | 1.4 | 1.7 | 2.1 |
| Land Use and Cover | Pasture | 100 | 32.3 | 1.2 | 9.0 | 1.6 | 3.2 | 2.1 | 9.0 | 1.8 |
| Land Use and Cover | Pasture | 1000 | 1.8 | 0.9 | 3.2 | 1.4 | 1.7 | 2.2 | 1.8 | 0.9 |
| Land Use and Cover | Primary land | 10 | 1.4 | 1.7 | 1.9 | 0.9 | 0.4 | 2.8 | 4.6 | 3.8 |
| Land Use and Cover | Primary land | 100 | 1.1 | 1.9 | 1.5 | 0.5 | 0.2 | 3.5 | 3.5 | 2.3 |
| Land Use and Cover | Primary land | 1000 | 1.3 | 0.4 | 2.3 | 0.3 | 0.3 | 3.3 | 2.1 | 1.0 |
| Land Use and Cover | Secondary land | 10 | 1.3 | 2.8 | 0.7 | 13.3 | 4.6 | 3.5 | 0.8 | 5.3 |
| Land Use and Cover | Secondary land | 100 | 2.8 | 4.6 | 2.8 | 3.0 | 3.2 | 4.3 | 1.0 | 3.2 |
| Land Use and Cover | Secondary land | 1000 | 0.8 | 2.8 | 1.0 | 3.5 | 1.1 | 3.3 | 0.8 | 5.7 |
| Land Use and Cover | Urban land | 10 | 4.9 | 5.7 | 3.3 | 6.7 | 3.0 | 1.2 | 1.1 | 1.3 |
| Land Use and Cover | Urban land | 100 | 1.7 | 0.7 | 1.2 | 0.3 | 3.8 | 0.1 | 0.5 | 0.6 |
| Land Use and Cover | Urban land | 1000 | 0.4 | 0.2 | 0.4 | 0.3 | 9.0 | 0.1 | 0.3 | 0.1 |
| Land Use and Cover | Cultivated and Managed Vegetation | 10 | 6.1 | 1.4 | 13.3 | 3.3 | 3.0 | 1.3 | 1.5 | 1.7 |
| Land Use and Cover | Cultivated and Managed Vegetation | 100 | 1.0 | 1.3 | 3.0 | 2.3 | 1.9 | 1.5 | 0.8 | 1.5 |
| Land Use and Cover | Cultivated and Managed Vegetation | 1000 | 0.5 | 1.6 | 0.3 | 2.1 | 1.1 | 1.1 | 0.4 | 1.0 |
| Land Use and Cover | Regularly Flooded Vegetation | 10 | 1.9 | 2.8 | 1.2 | 1.9 | 1.3 | 0.5 | 0.8 | 0.9 |
| Land Use and Cover | Regularly Flooded Vegetation | 100 | 2.8 | 3.0 | 1.7 | 1.8 | 2.0 | 0.9 | 1.4 | 1.0 |
| Land Use and Cover | Regularly Flooded Vegetation | 1000 | 3.5 | 2.1 | 2.1 | 3.5 | 4.0 | 1.4 | 1.8 | 1.6 |
| Land Use and Cover | Herbaceous Vegetation | 10 | 4.3 | 1.1 | 6.7 | 0.4 | 1.3 | 1.0 | 2.0 | 2.3 |
| Land Use and Cover | Herbaceous Vegetation | 100 | 2.1 | 1.0 | 0.4 | 0.2 | 2.1 | 1.6 | 1.2 | 5.7 |
| Land Use and Cover | Herbaceous Vegetation | 1000 | 1.6 | 0.5 | 0.2 | 0.2 | 1.4 | 1.2 | 0.7 | 1.1 |
| Land Use and Cover | Open water | 10 | 6.7 | 15.7 | 5.7 | 8.1 | 1.9 | 4.0 | 1.9 | 1.6 |
| Land Use and Cover | Open water | 100 | 3.0 | 0.6 | 5.3 | 0.6 | 3.0 | 1.1 | 1.4 | 1.0 |
| Land Use and Cover | Open water | 1000 | 2.3 | 0.4 | 4.3 | 0.1 | 5.3 | 0.7 | 1.3 | 0.9 |
| Land Use and Cover | Lake_river_reservoir | 10 | 0.9 | 1.2 | 1.4 | 0.5 | 1.2 | 0.6 | 1.4 | 0.9 |
| Land Use and Cover | Lake_river_reservoir | 100 | 1.2 | 0.9 | 1.5 | 0.8 | 1.6 | 1.3 | 0.8 | 0.4 |
| Land Use and Cover | Lake_river_reservoir | 1000 | 1.0 | 0.8 | 1.7 | 0.8 | 1.1 | 1.1 | 1.4 | 0.5 |
| Land Use and Cover | Wetland_combine | 10 | 0.5 | 0.6 | 0.9 | 0.2 | 0.6 | 0.3 | 0.7 | 0.6 |
| Land Use and Cover | Wetland_combine | 100 | 0.4 | 0.1 | 1.4 | 0.0 | 0.5 | 0.0 | 0.6 | 0.4 |
| Land Use and Cover | Wetland_combine | 1000 | 0.3 | 0.4 | 1.0 | 0.3 | 0.5 | 0.4 | 0.5 | 0.5 |
| Land Use and Cover | Wetland_other | 10 | 0.3 | 0.9 | 0.7 | 0.0 | 0.4 | 0.2 | 0.4 | 0.9 |
| Land Use and Cover | Wetland_other | 100 | 0.2 | 0.0 | 0.7 | 0.0 | 0.3 | 0.1 | 0.4 | 0.2 |
| Land Use and Cover | Wetland_other | 1000 | 0.4 | 0.3 | 0.6 | 0.1 | 0.3 | 0.2 | 0.3 | 0.2 |
| Land Use and Cover | Wetland concentration | 10 | 8.1 | 7.3 | 4.3 | 3.2 | 2.8 | 2.0 | 5.3 | 4.3 |
| Land Use and Cover | Wetland concentration | 100 | 6.7 | 6.1 | 4.9 | 2.7 | 1.7 | 2.8 | 4.6 | 4.0 |
| Land Use and Cover | Wetland concentration | 1000 | 3.0 | 1.8 | 3.8 | 0.6 | 1.4 | 0.5 | 5.3 | 1.8 |
| Topography | Elevation | 10 | 0.6 | 2.8 | 0.4 | 6.1 | 0.5 | 7.4 | 0.8 | 3.8 |
| Topography | Elevation | 100 | 0.8 | 4.6 | 0.1 | 1.6 | 0.3 | 6.1 | 1.3 | 3.2 |
| Topography | Elevation | 1000 | 0.7 | 4.9 | 0.1 | 2.4 | 0.5 | 5.7 | 0.8 | 2.6 |
| Socio-economic | GDP | 10 | 11.5 | 3.6 | 14.0 | 3.0 | 3.3 | 3.7 | 3.8 | 3.5 |
| Socio-economic | GDP | 100 | 7.3 | 3.0 | 13.3 | 1.6 | 6.7 | 0.9 | 4.6 | 3.3 |
| Socio-economic | GDP | 1000 | 1.7 | 0.0 | 1.0 | 0.0 | 8.1 | 0.0 | 2.2 | 0.3 |
| Socio-economic | Human population | 10 | 1.9 | 2.4 | 4.0 | 4.9 | 1.3 | 0.8 | 0.9 | 0.5 |
| Socio-economic | Human population | 100 | 1.4 | 1.9 | 2.2 | 4.0 | 3.5 | 0.1 | 0.9 | 0.4 |
| Socio-economic | Human population | 1000 | 1.3 | 0.3 | 1.1 | 0.2 | 5.7 | 0.3 | 0.9 | 0.3 |
| Biodiversity | Livestock count | 10 | 13.3 | 6.1 | 13.3 | 10.1 | 2.2 | 1.2 | 5.3 | 2.6 |
| Biodiversity | Livestock count | 100 | 5.3 | 1.9 | 24.0 | 0.5 | 2.3 | 0.1 | 10.1 | 1.1 |
| Biodiversity | Livestock count | 1000 | 4.6 | 0.3 | 32.3 | 0.1 | 3.5 | 0.1 | 8.1 | 0.3 |
| Biodiversity | Mammal species richness | 10 | 9.0 | 6.7 | 4.0 | 15.7 | 1.8 | 1.7 | 1.1 | 3.0 |
| Biodiversity | Mammal species richness | 100 | 24.0 | 8.1 | 3.5 | 13.3 | 2.6 | 1.9 | 2.8 | 2.1 |
| Biodiversity | Mammal species richness | 1000 | 13.3 | 5.3 | 2.7 | 11.5 | 2.0 | 2.3 | 3.3 | 2.7 |
| Biodiversity | Flyway_Anseriformes | 10 | 0.2 | 0.0 | 2.6 | 0.1 | 0.2 | 0.1 | 1.0 | 1.3 |
| Biodiversity | Flyway_Anseriformes | 100 | 0.1 | 0.9 | 0.9 | 0.0 | 0.0 | 0.1 | 1.1 | 2.0 |
| Biodiversity | Flyway_Anseriformes | 1000 | 0.0 | 2.7 | 0.8 | 0.1 | 0.1 | 0.4 | 0.9 | 1.7 |
| Biodiversity | Flyway_Apodiformes | 10 | 0.1 | 2.4 | 0.1 | 0.8 | 0.2 | 1.7 | 0.9 | 1.2 |
| Biodiversity | Flyway_Apodiformes | 100 | 0.1 | 1.0 | 0.0 | 0.4 | 0.3 | 1.1 | 1.3 | 1.6 |
| Biodiversity | Flyway_Apodiformes | 1000 | 0.1 | 1.1 | 0.0 | 0.2 | 0.3 | 1.2 | 1.4 | 0.8 |
| Biodiversity | Flyway_Passeriformes | 10 | 9.0 | 0.3 | 1.0 | 0.3 | 1.7 | 0.2 | 0.4 | 2.0 |
| Biodiversity | Flyway_Passeriformes | 100 | 3.2 | 0.2 | 2.4 | 0.4 | 0.9 | 0.2 | 0.5 | 2.1 |
| Biodiversity | Flyway_Passeriformes | 1000 | 2.4 | 0.2 | 4.0 | 0.5 | 1.0 | 1.0 | 0.4 | 1.9 |
| Biodiversity | Birds Directive | 10 | 1.6 | 0.9 | 0.1 | 0.1 | 3.0 | 1.1 | 2.7 | 2.5 |
| Biodiversity | Birds Directive | 100 | 2.1 | 0.6 | 0.1 | 0.2 | 2.4 | 0.5 | 2.6 | 2.6 |
| Biodiversity | Birds Directive | 1000 | 2.3 | 0.6 | 0.1 | 0.3 | 3.2 | 0.5 | 2.4 | 2.6 |
| Biodiversity | Birds and Habitats Directives | 10 | 0.8 | 1.1 | 0.1 | 0.1 | 3.0 | 1.4 | 2.1 | 1.4 |
| Biodiversity | Birds and Habitats Directives | 100 | 0.8 | 1.0 | 0.0 | 0.5 | 1.6 | 0.6 | 2.2 | 0.6 |
| Biodiversity | Birds and Habitats Directives | 1000 | 0.7 | 2.6 | 0.1 | 2.8 | 1.7 | 1.9 | 2.6 | 1.9 |
| Biodiversity | Habitats Directive | 10 | 1.6 | 0.8 | 0.1 | 0.1 | 3.8 | 1.2 | 2.8 | 2.7 |
| Biodiversity | Habitats Directive | 100 | 2.0 | 0.6 | 0.1 | 0.2 | 4.0 | 0.5 | 2.5 | 1.3 |
| Biodiversity | Habitats Directive | 1000 | 2.6 | 0.5 | 0.1 | 0.2 | 3.8 | 0.5 | 2.6 | 1.0 |
| Biodiversity | Richness of forest-related species and habitats | 10 | 4.3 | 3.3 | 2.4 | 2.7 | 1.1 | 0.5 | 2.0 | 2.4 |
| Biodiversity | Richness of forest-related species and habitats | 100 | 2.1 | 3.1 | 2.2 | 2.1 | 0.0 | 0.4 | 2.2 | 2.2 |
| Biodiversity | Richness of forest-related species and habitats | 1000 | 1.1 | 3.0 | 2.1 | 2.0 | 0.0 | 0.3 | 2.1 | 2.3 |
| Biodiversity | Culex pipiens status | 10 | 2.7 | 2.7 | 3.5 | 2.8 | 0.4 | 0.7 | 1.6 | 1.8 |
| Biodiversity | Culex pipiens status | 100 | 2.7 | 3.3 | 3.2 | 3.0 | 0.6 | 0.4 | 1.9 | 1.8 |
| Biodiversity | Culex pipiens status | 1000 | 2.8 | 2.6 | 3.3 | 2.7 | 0.7 | 0.8 | 1.9 | 1.9 |

*Bayes factor (BF>20 in red suggests significant) supports for the association between dispersal durations and environmental distances (n=37) computed for each branch (resistance, conductance) in different scales (k=10,100,1000). Analyses were performed on data of NS3, NS5 and also separated for Cluster A and B separately. All factors with BF>20 have positive regression coefficient and with >90% positive value for Q statistics (details of Q statistics are shown in S1 Data).
