# Supplementary material for: West Nile virus spread in Europe: Phylogeographic pattern analysis and key drivers
Source: PLoS Pathog. 2024 Jan 25;20(1):e1011880. doi: 10.1371/journal.ppat.1011880 (PMC10810478; doi:10.1371/journal.ppat.1011880)
Supplement: S1 Fig — The Definition for each predictor is shown in the (S2 Table). Data were log-transformed where necessary for better visualization. The unit of each predictor is shown after the predictor name above each panel. The European shapefile was created using the R package “rworldmap” (https://cran.r-project.org/web/packages/rworldmap/). (DOCX) [file ppat.1011880.s009.docx]

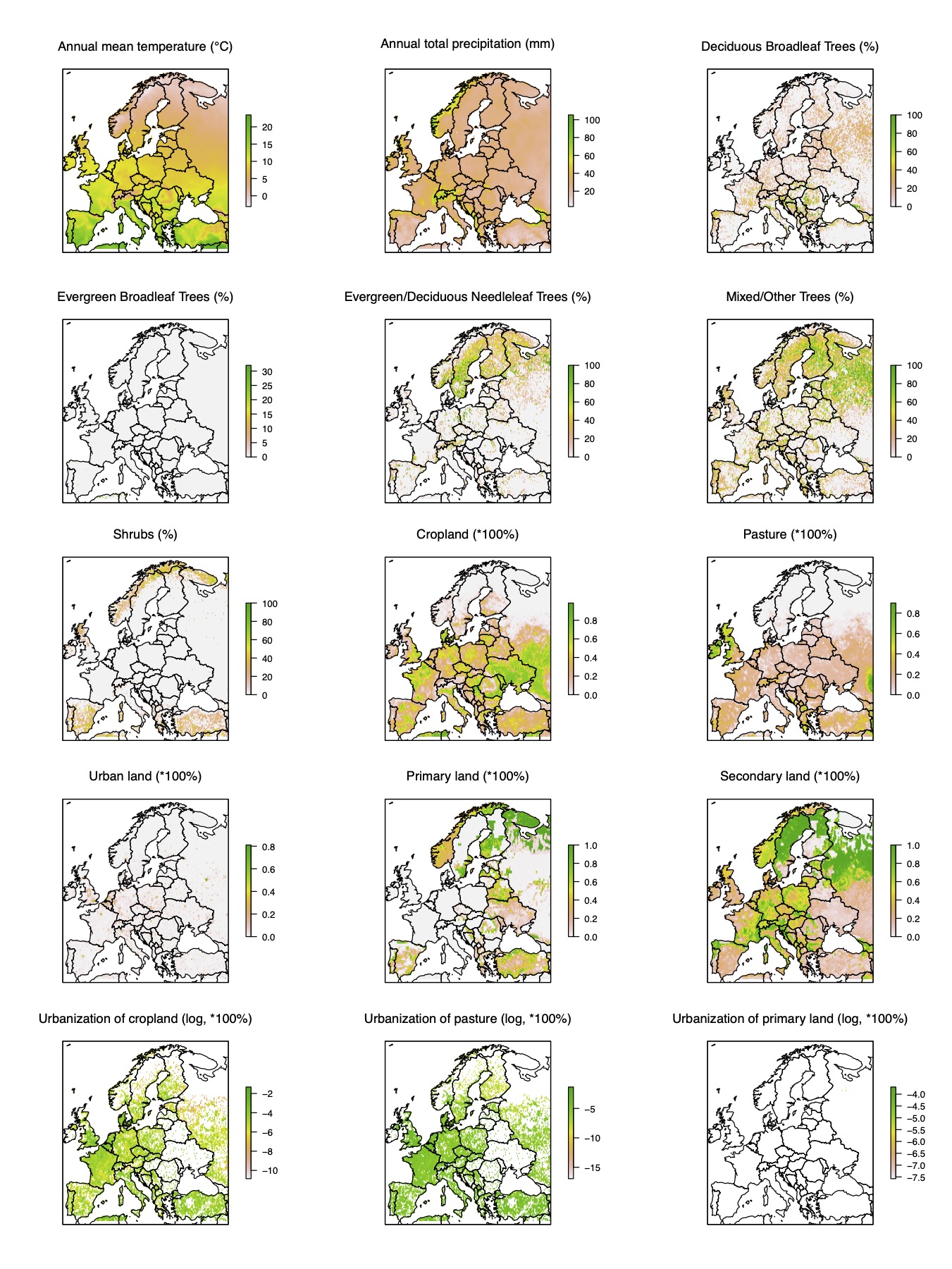


# S1 Figure: Distribution of predictors for dispersal of West Nile virus in Europe.

**Definition for each predictor is shown in the S2 Table. Data were log-transformed where necessary for better visualization. Unit of each predictor is shown after the predictor name above each panel. The European shapefile was created using the R package “rworldmap” (**[**https://cran.r-project.org/web/packages/rworldmap/**](https://cran.r-project.org/web/packages/rworldmap/)**).**


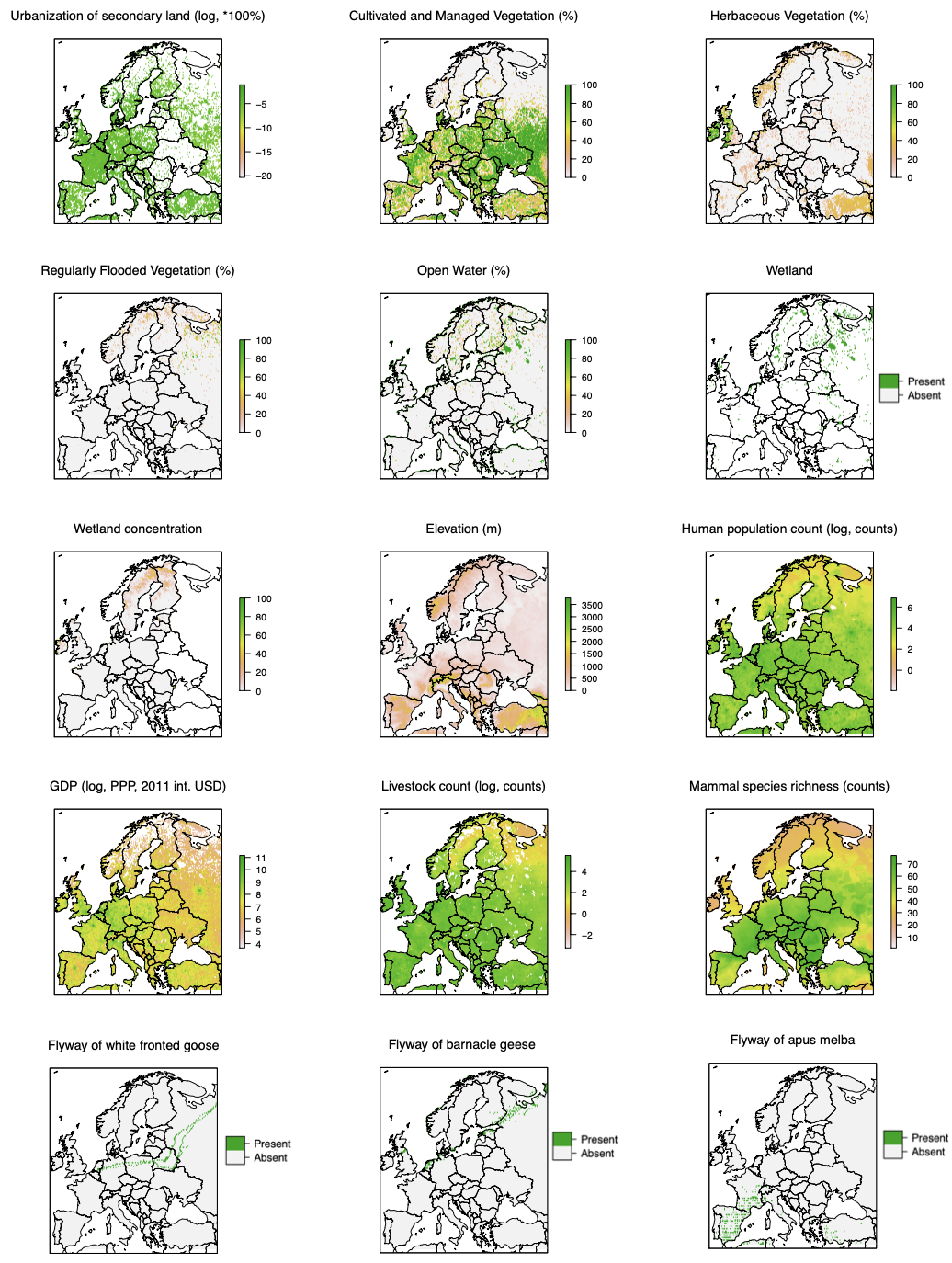


**S1 Fig continued - 1**


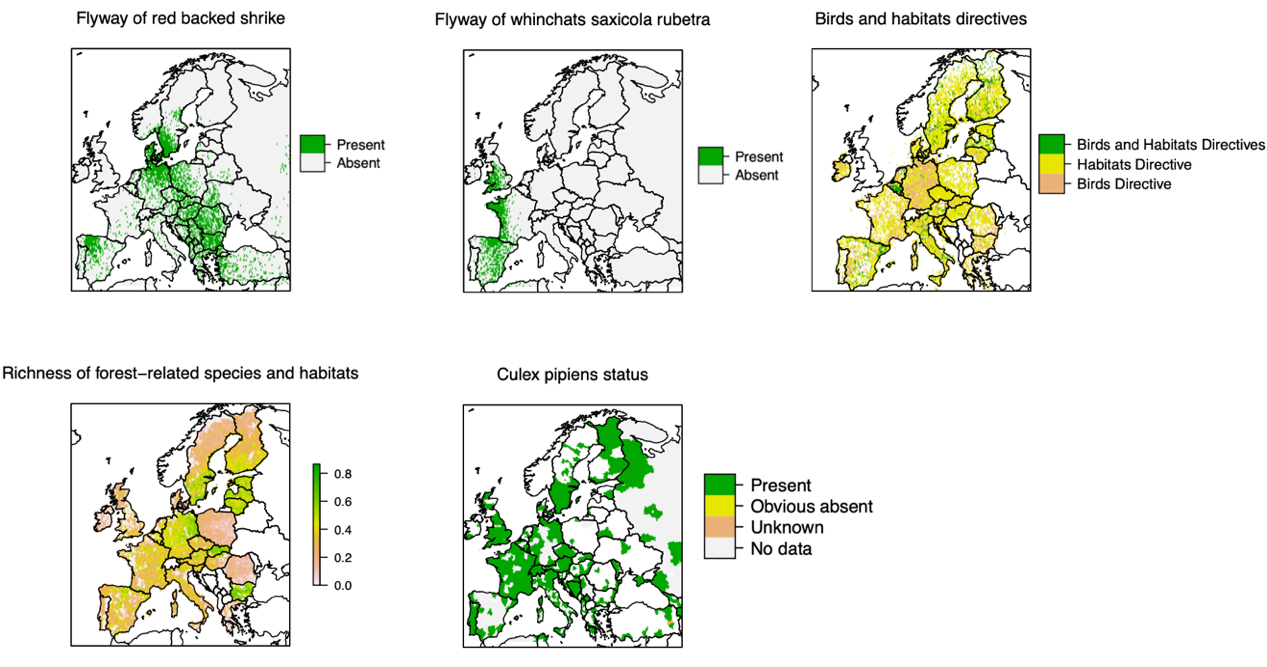


**S1 Fig continued - 2**
