# Supplementary material for: West Nile virus spread in Europe: Phylogeographic pattern analysis and key drivers
Source: PLoS Pathog. 2024 Jan 25;20(1):e1011880. doi: 10.1371/journal.ppat.1011880 (PMC10810478; doi:10.1371/journal.ppat.1011880)
Supplement: S2 Fig — Data in four months (January, April, July, and October) were shown for each predictor. Definition for each predictor is shown in the (S3 Table). The unit of each predictor is shown after the predictor name above each panel. The European shapefile was created using the R package “rworldmap” (https://cran.r-project.org/web/packages/rworldmap/). (DOCX) [file ppat.1011880.s010.docx]

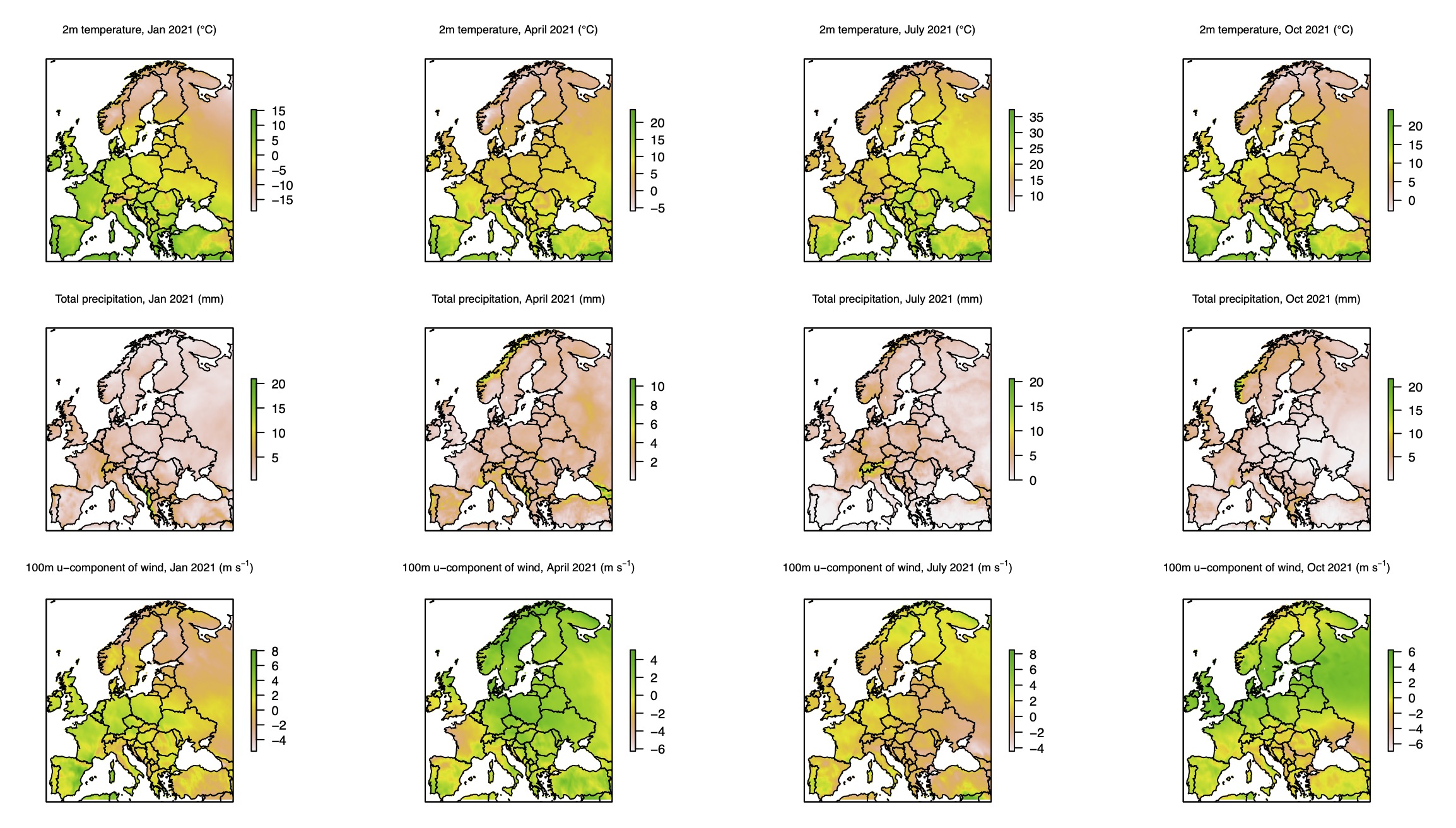


# S2 Fig: Distribution of predictors for viral genetic diversity over time.

**Data in four months (January, April, July, and October) were shown for each predictor. Definition for each predictor is shown in S3 Table**. **Unit of each predictor is shown after the predictor name above each panel. The European shapefile was created using the R package “rworldmap” (https://cran.r-project.org/web/packages/rworldmap/).**


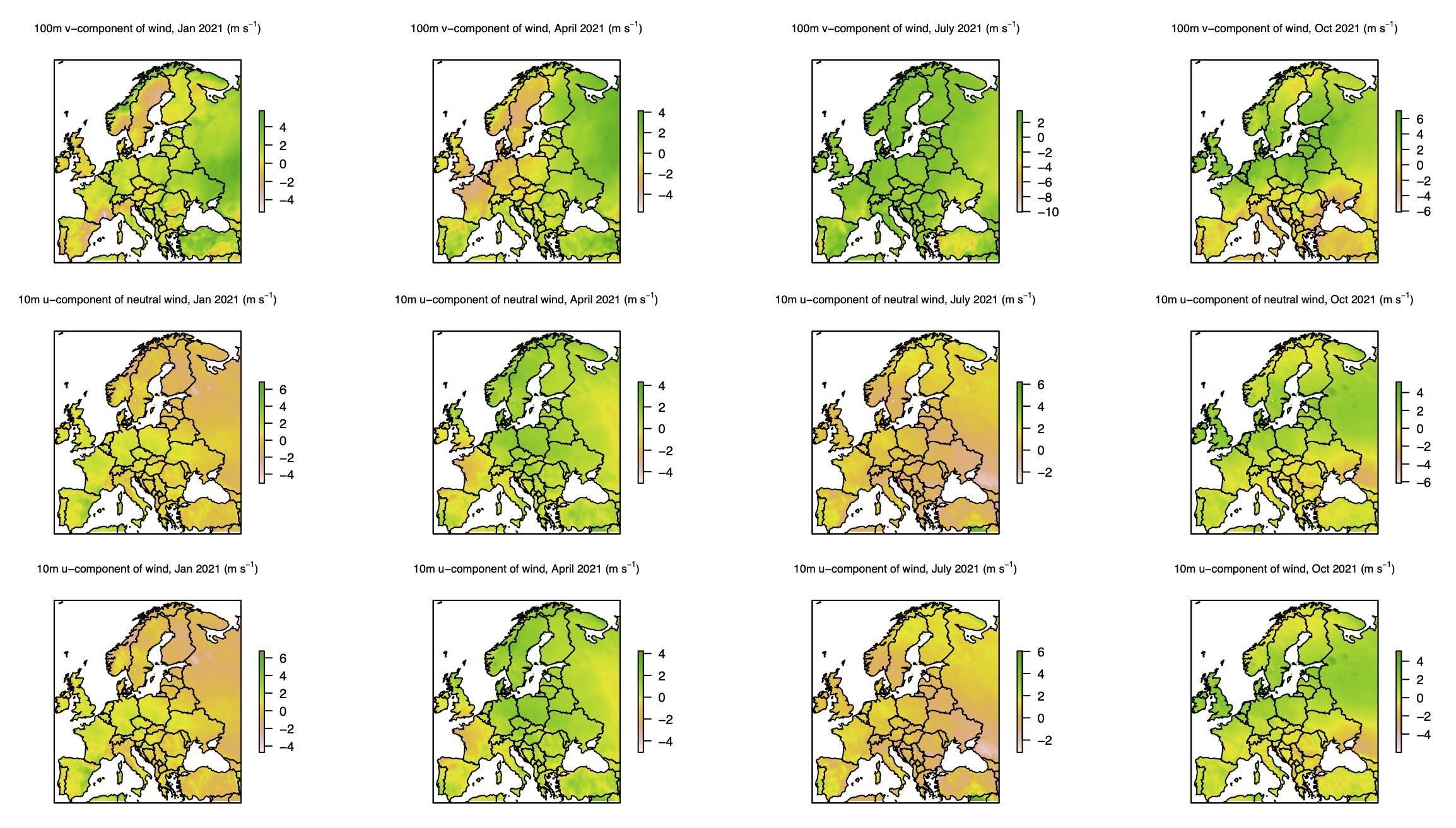


**S2 Fig continued - 1**


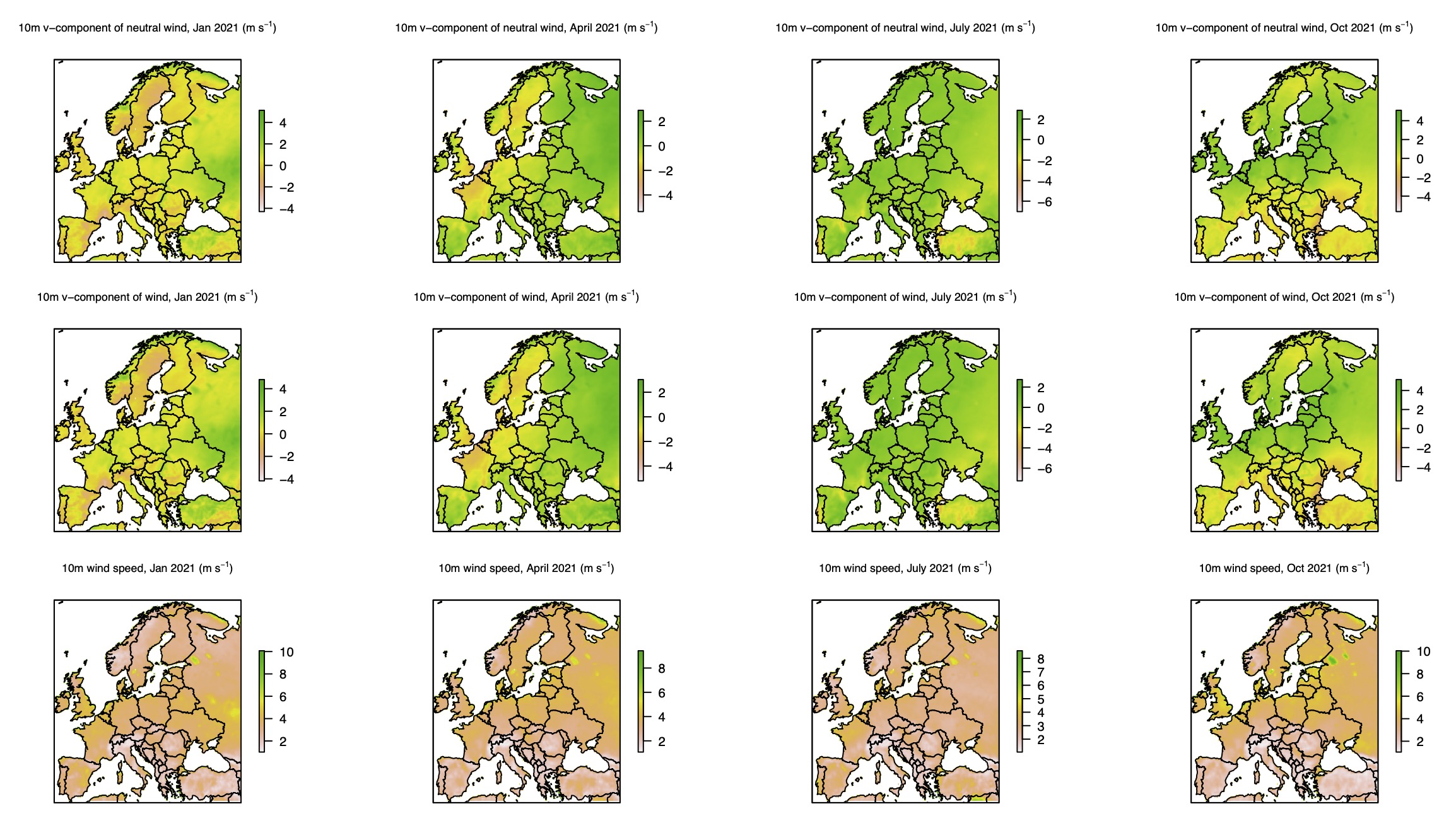


**S2 Fig continued - 2**


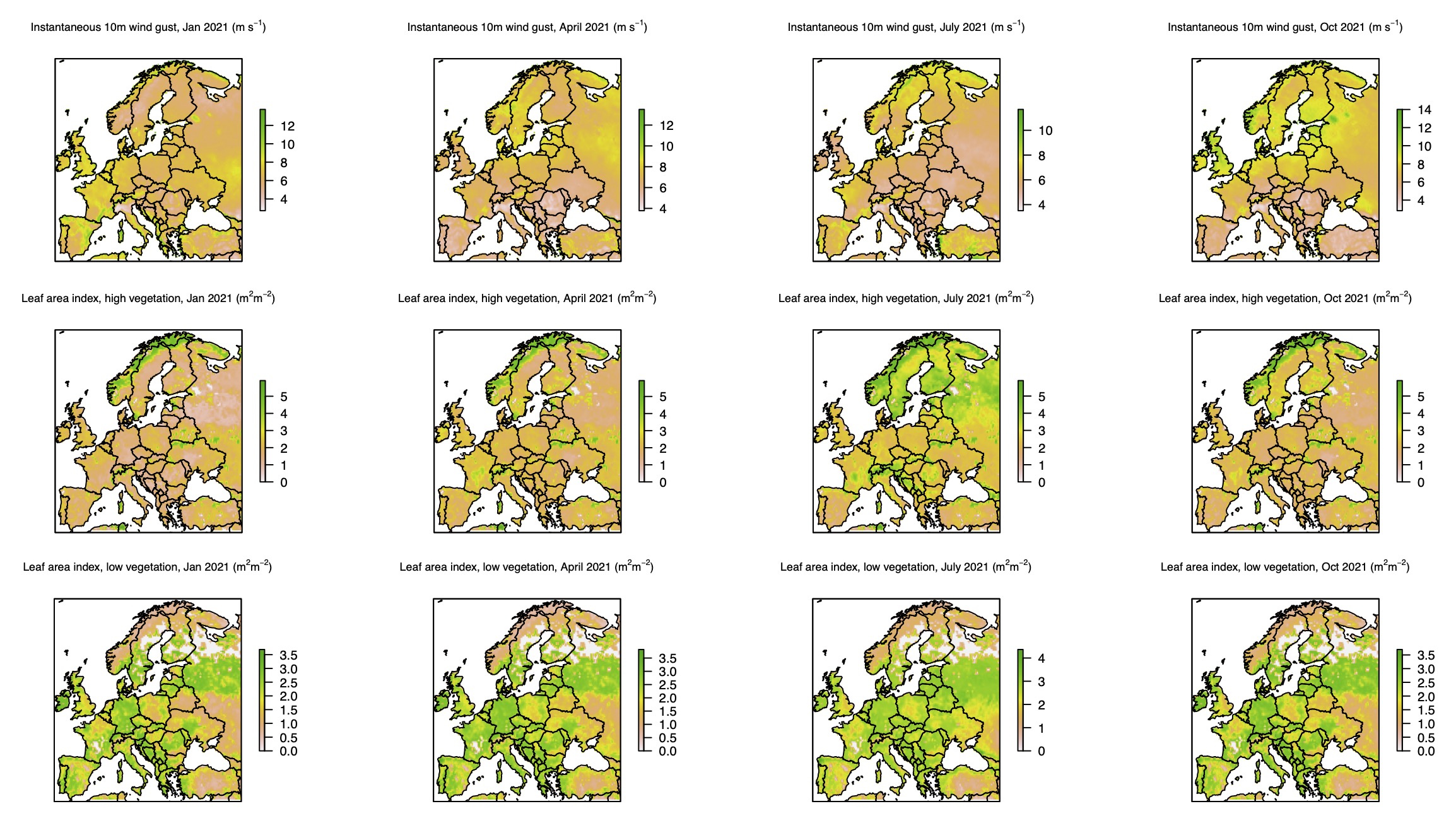


**S2 Fig continued - 3**
