# Supplementary material for: West Nile virus spread in Europe: Phylogeographic pattern analysis and key drivers
Source: PLoS Pathog. 2024 Jan 25;20(1):e1011880. doi: 10.1371/journal.ppat.1011880 (PMC10810478; doi:10.1371/journal.ppat.1011880)
Supplement: S3 Fig — (a) Comparison between cumulative human cases reported by ECDC (between 2008–2021, total n = 4188) and the number of WNV sequences (between 1971–2021, total n = 485) isolated from 22 different countries. (b) The sequencing effort (ratio of the number of sequences available to the number of human cases reported) per country is shown on the map: red from light to dark indicates the ratio from low to high; green indicates no sequence available although human cases have been reported; grey indicated neither human cases nor sequences are available. The European shapefile was created using the R package “raster” (https://cran.r-project.org/web/packages/raster/). (DOCX) [file ppat.1011880.s011.docx]

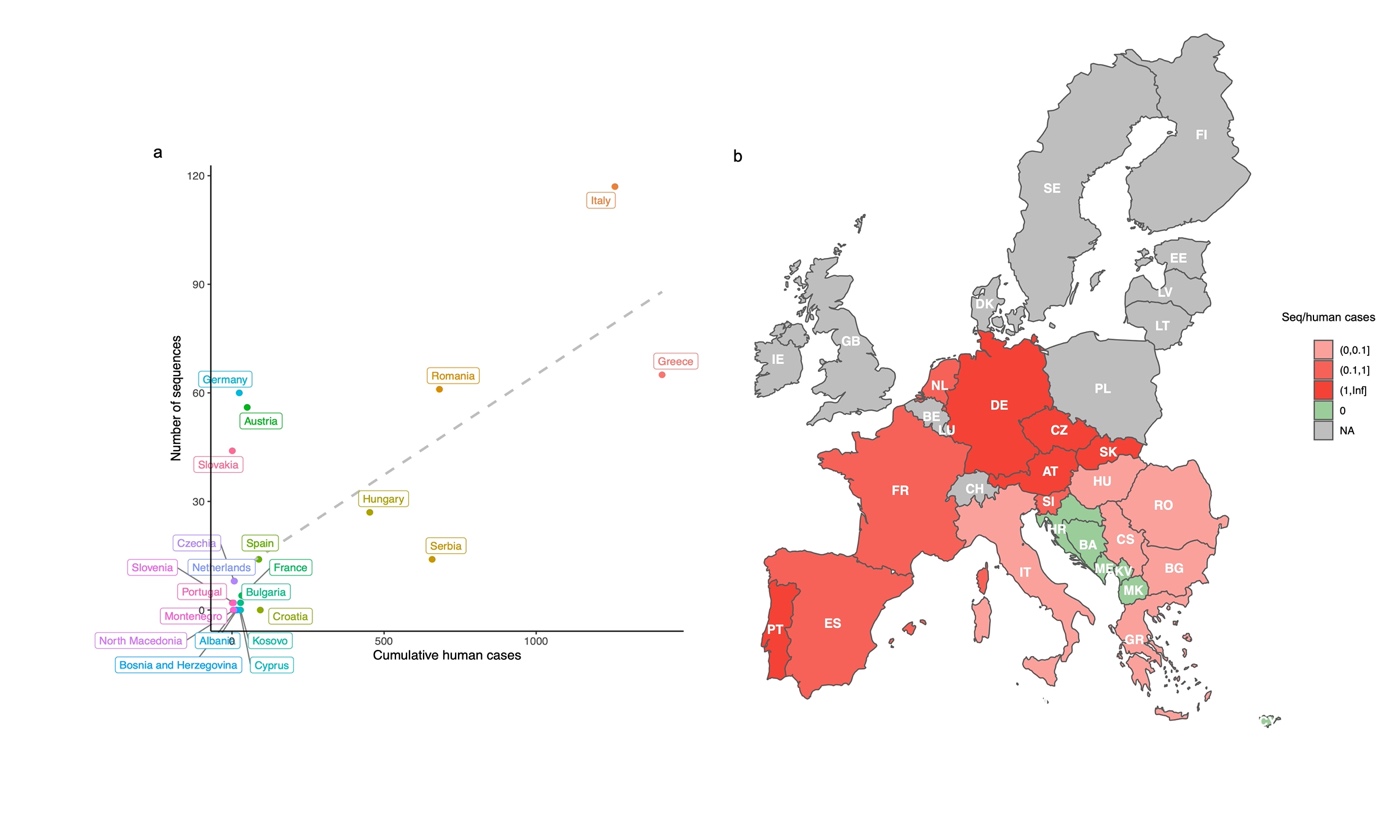


# S3 Fig: Surveillance and sequencing effort of WNV in Europe

**(a) Comparison between cumulative human cases reported by ECDC (between 2008-2021, total n=4188) and the number of WNV sequences (between 1971-2021, total n=485) isolated from 22 different countries. (b) The sequencing effort (ratio of the number of sequences available to the number of human cases reported) per country is shown on the map: red from light to dark indicated the ratio from low to high; green indicated no sequence available although human cases have been reported; grey indicated neither human cases nor sequences are available. The European shapefile was created using the R package “raster” (https://cran.r-project.org/web/packages/raster/).**
