# Supplementary material for: West Nile virus spread in Europe: Phylogeographic pattern analysis and key drivers
Source: PLoS Pathog. 2024 Jan 25;20(1):e1011880. doi: 10.1371/journal.ppat.1011880 (PMC10810478; doi:10.1371/journal.ppat.1011880)
Supplement: S5 Fig — (a) Sequences from different hosts on maps. The colors for hosts are consistent in a-d. (b) Relative frequencies of sequences sampled from 5 different hosts. (c) Relative frequencies of sequences sampled from different bird orders. (d) Relative frequencies of sequences sampled from different mosquito species. The European shapefile was created using the R package “maps” (https://cran.r-project.org/web/packages/maps/). (DOCX) [file ppat.1011880.s013.docx]

# S5 Fig: Distributions of WNV-2a sequences from different hosts

**(a) Sequences from different hosts on maps. The colors for hosts are consistent in a-d. (b) Relative frequencies of sequences sampled from 5 different hosts. (c) Relative frequencies of sequences sampled from different bird orders. (d) Relative frequencies of sequences sampled from different mosquito species. The European shapefile was created using the R package “maps” (https://cran.r-project.org/web/packages/maps/).**
