# Supplementary material for: West Nile virus spread in Europe: Phylogeographic pattern analysis and key drivers
Source: PLoS Pathog. 2024 Jan 25;20(1):e1011880. doi: 10.1371/journal.ppat.1011880 (PMC10810478; doi:10.1371/journal.ppat.1011880)
Supplement: S6 Fig — Trees were reconstructed using sequences of ns3 gene (a) and ns5 gene (b) mapping with countries (n = 14). (DOCX) [file ppat.1011880.s014.docx]

# S6 Fig: Time-scaled MCC trees of WNV mapping with countries

**Trees were reconstructed using sequences of ns3 gene (a) and ns5 gene (b) mapping with countries (n=14).**
