# Supplementary material for: West Nile virus spread in Europe: Phylogeographic pattern analysis and key drivers
Source: PLoS Pathog. 2024 Jan 25;20(1):e1011880. doi: 10.1371/journal.ppat.1011880 (PMC10810478; doi:10.1371/journal.ppat.1011880)
Supplement: S7 Fig — Transmission networks inferred from the joint analysis of ns3 and ns5 phylogenies of WNV-2a sequences. (a) between European countries. (b) between regions in Greece. The size of node indicates the number of samples; edge weight indicates the median number of transmissions between pairs of locations; the arrow on edge indicates transmission direction; color of the edge from light to dark indicates Bayes Factor (BF) support from low to high only transmissions with BF >5 are shown). The correlated farms are grouped together. Nodes with no link to the others indicated no significant transmissions with other areas although sequences have been sampled. (DOCX) [file ppat.1011880.s015.docx]

# S7 Fig: Quantified WNV transmission networks

**Transmission networks inferred from the joint analysis of ns3 and ns5 phylogenies of WNV-2a sequences. (a) between European countries. (b) between regions in Greece. Size of node indicates number of samples; edge weight indicates median number of transmissions between pairs of locations; arrow on edge indicates transmission direction; color of edge from light to dark indicates Bayes Factor (BF) support from low to high only transmissions with BF >5 are shown). The correlated farms are grouped together. Nodes with no link to the others indicated no significant transmissions with other areas although sequences have been sampled.**
