# Supplementary material for: West Nile virus spread in Europe: Phylogeographic pattern analysis and key drivers
Source: PLoS Pathog. 2024 Jan 25;20(1):e1011880. doi: 10.1371/journal.ppat.1011880 (PMC10810478; doi:10.1371/journal.ppat.1011880)
Supplement: S8 Fig — a) ns3 gene, b) ns5 gene, c) full genomes and d) the number of sequences for each dataset. The European shapefile was created using the R package “maps” (https://cran.r-project.org/web/packages/maps/). (DOCX) [file ppat.1011880.s016.docx]

# S8 Fig: Geographic distributions of WNV-2a sequences dataset

**a) ns3 gene, b) ns5 gene and c) full genomes and d) the number of sequences for each dataset. The European shapefile was created using the R package “maps” (https://cran.r-project.org/web/packages/maps/).**
